# Scymicrosin_7–26_, a *Scylla paramamosain*-derived novel antimicrobial peptide, exhibits efficacy against multidrug-resistant ESKAPE pathogens and anti-inflammatory activity

**DOI:** 10.3389/fmicb.2025.1732053

**Published:** 2025-12-17

**Authors:** Cong Hu, Fangyi Chen, Ying Zhou, Ting Yang, Kejian Wang, Sheng Yang, Xiangqi Chen

**Affiliations:** 1 Department of Pulmonary and Critical Care Medicine, Fujian Medical University Union Hospital, Fuzhou, China; 2 State-Province Joint Engineering Laboratory of Marine Bioproducts and Technology, College of Ocean and Earth Sciences, Xiamen University, Xiamen, China; 3 State Key Laboratory of Marine Environmental Science, College of Ocean and Earth Sciences, Xiamen University, Xiamen, China; 4 Innovation Research Institute for Marine Biological Antimicrobial Peptide Industry Technology, Fujian Ocean Innovation Center, Xiamen, China; 5 The Second Affiliated Hospital of Fujian University of Traditional Chinese Medicine, Fuzhou, China; 6 Department of Oncology, Fujian Medical University Union Hospital, Fuzhou, China; 7 NHC Key Laboratory of Etiological Epidemiology of Chronic Diseases with High Incidence in Fujian-Taiwan Area (Co-Construction), Fujian Medical University, Fuzhou, China

**Keywords:** antimicrobial peptide, multidrug-resistant bacteria, antimicrobial mechanisms, anti-inflammatory, signaling pathways

## Abstract

The escalating misuse of antibiotics has precipitated a worldwide crisis of bacterial resistance, greatly complicating the clinical management of multidrug-resistant bacterial infections, which now present a profound threat and a growing burden on public health systems. This situation necessitates the development of innovative anti-infective therapeutics. This work focuses on Scymicrosin_7–26_, a newly identified antimicrobial peptide (AMP) sourced from the crustacean *Scylla paramamosain*. AMPs typically derived from crustaceans are often characterized by suboptimal potency, instability, potential toxicity, and a narrow spectrum of activity, whereas Scymicrosin_7–26_ exhibits certain improvements in these regards. It exhibited antibacterial activity against five types of common clinically isolated multidrug-resistant organisms (MDROs). It inhibited both the formation and maturation of biofilms in carbapenem-resistant *Pseudomonas aeruginosa* (CR-PA) as well as methicillin-resistant *Staphylococcus aureus* (MRSA) without readily inducing resistance. Scymicrosin_7–26_ retained stable antimicrobial activity under physiological salt conditions and showed no significant antagonism when combined with several conventional antibiotics. It also demonstrated low toxicity toward RAW264.7, HEK293T, and Beas-2B cell lines, as well as human erythrocytes. Using fluorescence and electron microscopy, we observed disruption of bacterial surface structures. DNA binding assays further indicated the peptide’s capacity to interact with bacterial genomic DNA. Moreover, Scymicrosin_7–26_ alleviated lipopolysaccharide (LPS)-triggered inflammatory responses via concurrent blockade of MAPK and NF-κB pathway activation. With its antibacterial activity against multidrug-resistant pathogens, anti-inflammatory property, and safety profile, Scymicrosin_7–26_ exhibits therapeutic potential for managing infections caused by multidrug-resistant bacteria.

## Graphical abstract

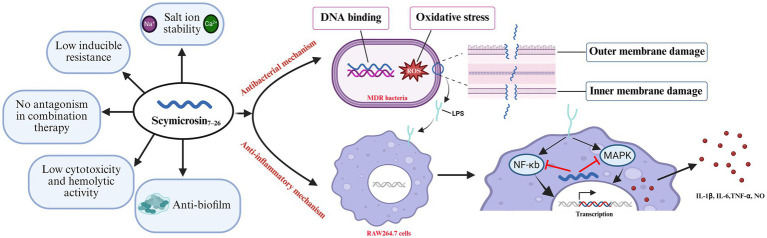

## 1 Introduction

The identification of penicillin by Alexander Fleming in 1928 marked the beginning of a new era in which antibiotics have dramatically curtailed fatalities from infectious diseases (Mohr, 2016). Over the past century, these agents have undergone multiple cycles of development and clinical deployment (Ventola, 2015; Aslam et al., 2018). However, the rising incidence of inappropriate antibiotic use in clinical practice has accelerated the emergence of resistant pathogens (English and Gaur, 2010). Projections indicate that by 2050, drug-resistant infections will claim 10 million lives annually worldwide (Guryanova and Khaitov, 2021; Sunuwar and Azad, 2021), becoming a leading cause of global mortality (Jiang et al., 2024). Multidrug-resistant bacteria (MDR bacteria), defined as those that are non-susceptible to at least one agent in three or more antimicrobial categories (Magiorakos et al., 2012). Among them, the ESKAPE pathogens—*Enterococcus faecium*, *Staphylococcus aureus*, *Klebsiella pneumoniae*, *Acinetobacter baumannii*, *Pseudomonas aeruginosa*, and *Enterobacter* spp.—are frequently associated with high levels of multidrug resistance (Namukonda et al., 2025; Seid et al., 2025). These organisms account for a significant proportion of both hospital- and community-acquired infections, resulting in clinical manifestations including pneumonia, urinary tract infections, and intensive care unit (ICU)-related complications (Deng et al., 2022; Teng et al., 2023; El Husseini et al., 2024), and are associated with considerable mortality worldwide (Hong et al., 2021; Teng et al., 2023; Kramarska et al., 2024).

The respiratory tract is one of the most frequent sites of ESKAPE pathogen colonization and infection, with nosocomial pneumonia, respiratory infections associated with mechanical ventilation, and infected bronchiectasis representing common clinical manifestations (Zhang et al., 2020; Mayor et al., 2021). Current conventional antibiotic therapies face considerable challenges in this context. Agents such as polymyxins, tigecycline, and beta-lactam combination regimens are often limited by adverse effects and the risk of inducing further resistance, underscoring the urgent need for novel antimicrobial strategies.

Antimicrobial peptides (AMPs), key mediators of innate host defense mechanisms found across animals, plants, and bacteria, represent a promising alternative (McMillan and Coombs, 2020; Shwaiki et al., 2022; George and Orlando, 2023). They exhibit broad-spectrum activity against bacteria (Ahmed et al., 2024), viruses (Chianese et al., 2022), fungi (Guerra et al., 2024), and parasites (Periwal et al., 2024), in addition to anti-inflammatory properties (Zhuo et al., 2022). Their antimicrobial mechanisms have attracted significant research interest in recent years.

The Antimicrobial Peptide Database (APD3) catalogs 5,680 peptides, including 3,351 natural, 1,733 synthetic, and 329 predicted AMPs. Of the 680 AMPs from arthropods, 76 are of crustacean origin. These crustacean-derived peptides are essential elements of the innate immune system, providing broad defense against pathogens in the absence of adaptive immunity (Zanjani et al., 2018). Found in marine arthropods such as shrimp and crabs, they are characterized by unique structures and mechanisms of action (Saucedo-Vázquez et al., 2022). However, natural AMPs often suffer from drawbacks such as cytotoxicity, hemolytic activity, and salt sensitivity. To address these limitations, researchers have employed chemical modification, genetic engineering, and advanced delivery systems to optimize lead compounds—aiming to retain antimicrobial potency while improving safety and stability.

Scymicrosin_7–26_ is a novel marine-derived AMP identified from *Scylla paramamosain*. Previous studies have confirmed its effectiveness against Methicillin-Resistant *Staphylococcus aureus* (MRSA) (Zhou et al., 2025). To address the lack of research on its potential properties, this peptide was further investigated to provide a reference for future development.

This research preliminarily evaluates the antibacterial activity of Scymicrosin_7–26_ against clinically isolated multidrug-resistant bacteria, as well as its potential to mitigate lipopolysaccharide (LPS)-induced inflammation. The results may contribute to future exploration of therapeutic approaches against the increasing threat of multidrug-resistant bacterial infections in humans.

## 2 Materials and methods

### 2.1 Antimicrobial agents

The methodologies for tissue preparation, gene amplification, bioinformatics analysis, and peptide synthesis were performed as previously described (Zhou et al., 2025). A brief description follows: Scymicrosin_7–26_ and its FITC-conjugated derivative were custom-synthesized by GenScript (Nanjing, China) with >95% purity. The HPLC profile, mass spectrum, and certificate of analysis are provided in Supplementary Figures S1–S3, respectively. This novel truncated peptide from Scylla paramamosain (sequence: GARQLVRRIVPVVLGALSRL-NH₂) was designed with key parameters typical of antimicrobial peptides: a net charge of +4 and 52.6% hydrophobicity. Its antimicrobial domain was validated by the CAMPR3 database, with threshold scores exceeding 0.8. The peptide was dissolved in sterile ultra-pure water, aliquoted, and stored at −80 °C to prevent repeated freeze–thaw cycles. Tigecycline, polymyxin B, lysostaphin, vancomycin, imipenem, amikacin, and lincomycin were acquired from Solarbio Science & Technology Co., Ltd. (Beijing, China).

### 2.2 Strains and cultivation

A total of 137 multidrug-resistant clinical isolates from respiratory specimens were included in this study, comprising the following five categories: 18 methicillin-resistant *Staphylococcus aureus* (MRSA), 28 carbapenem-resistant *Acinetobacter baumannii* (CR-AB), 23 carbapenem-resistant *Klebsiella pneumoniae* (CR-KP), 22 carbapenem-resistant *Pseudomonas aeruginosa* (CR-PA), and 46 extended-spectrum *β*-lactamase-producing *Escherichia coli* (ESBL-EC) strains. All strains were provided by the Department of Laboratory Medicine, Fujian Medical University Union Hospital. Bacterial cultivation was carried out using Luria-Bertani (LB) broth. All experimental procedures strictly followed the biosafety guidelines and institutional safety regulations established by the source hospital.

### 2.3 Cell and cultivation

Three cell types (RAW264.7 murine macrophages, Beas-2B human lung epithelium, HEK293T human embryonic kidney cells) were cultivated in high-glucose Dulbecco s Modified Eagle Medium enriched with 10% FBS and 1% penicillin/streptomycin, and incubated at 37 °C with 5% CO₂. Cells were routinely passaged every 48 h.

### 2.4 Efficacy and safety profile of Scymicrosin_7–26_ against multidrug-resistant bacteria

#### 2.4.1 Antimicrobial susceptibility testing

Using the broth microdilution method in Müller-Hinton (MH) broth, we evaluated the minimum inhibitory concentration (MIC) of Scymicrosin_7–26_. Briefly, Scymicrosin_7–26_ was serially two-fold diluted in MH broth. Bacterial suspensions with a density of 1 × 10^6^ colony-forming units per milliliter (CFU/mL) were prepared using mid-logarithmic phase cultures. Each well of 96-well plates received 50 μL aliquots of both drug dilutions and bacterial suspensions. Wells containing MH broth with bacteria but no antimicrobial peptide served as the positive control, while wells containing only sterile MH broth were assigned as negative control. Following overnight incubation (16–18 h, 37 °C), the MIC was designated as the lowest concentration achieving complete inhibition of visual growth. MBC assessment involved subculturing from clear wells onto agar plates, with MBC defined as the minimum concentration demonstrating bactericidal activity (≥99.9% reduction) against the original inoculum (Huo et al., 2020).

#### 2.4.2 Selection criteria for experimental bacterial strains

A single isolate from each of the five clinical multidrug-resistant pathogens was selected for subsequent experiments. The MIC_50_ value, defined as the minimal concentration inhibiting 50% of strains, identifies isolates that balance susceptibility and resistance, thus representing a moderate resistance level within the population (Kowalska-Krochmal and Dudek-Wicher, 2021; García-Viñola et al., 2025). The screening procedure was as follows: Step 1: The minimum inhibitory concentration (MIC) of Scymicrosin_7–26_ against all isolates was determined, and the MIC₅₀ for each bacterial species was calculated. Step 2: Strains exhibiting MIC values equal to the MIC₅₀ of their respective species were identified, ensuring that the selected isolates demonstrated intermediate susceptibility to the antimicrobial peptide. Their antibiotic susceptibility profiles were then characterized (Supplementary Figures S4–S8). Step 3: Based on the susceptibility profiles, strains that exhibited predominant sensitivity to all tested antibiotics were prioritized as experimental isolates. If multiple strains met this criterion within a species, one was randomly chosen for further study.

#### 2.4.3 Growth inhibition assay

One representative strain from each of the five bacterial species—designated AB1 (CR-AB), KP1 (CR-KP), EC1 (ESBL-EC), PA1 (CR-PA), and MRSA1—was selected. Following an established protocol (Rao et al., 2021), each strain was diluted in MH broth to 1 × 10^6^ CFU/mL. Bacterial suspensions (50 μL) were exposed to 50 μL of Scymicrosin_7–26_ in 96-well plates, producing final concentrations of 0 (growth control), 0.5, and 1 × MIC. The starting OD₆₀₀ was measured immediately after mixing. Plates were maintained at 37 °C, and bacterial growth was assessed through OD₆₀₀ measurements at 2-h intervals until control wells reached mid-log phase. Established antibiotics (polymyxin B and vancomycin at 1 × MIC) were employed as positive controls, with all experimental conditions replicated three times.

#### 2.4.4 Time-killing curves

Bacterial strains were prepared at 1 × 10^6^ CFU/mL in fresh MH broth and treated with Scymicrosin_7–26_ to reach final concentrations of 0 (untreated control), 1, and 2 × MBC. Incubation was carried out at 37 °C with orbital shaking (190 rpm). At established time intervals, 100 μL samples were collected, diluted serially in 10-fold steps, and 50 μL of each dilution was spotted onto LB agar. After 24 h at 37 °C, viable bacteria were enumerated (Zhu et al., 2021). The results were plotted as survival rate versus time.

#### 2.4.5 Checkerboard assay

The combination effects of Scymicrosin_7–26_ with established antibiotics were evaluated via checkerboard microdilution assay (Riool et al., 2020). Bacterial suspensions (1 × 10^6^ CFU/mL) were inoculated into 96-well plates. Scymicrosin_7–26_ and the test antibiotic were serially diluted in two dimensions across the plate. After overnight incubation (16–18 h, 37 °C), the MICs of single agents and drug combinations were documented for the five test strains (AB1, KP1, EC1, PA1, MRSA1).

The fractional inhibitory concentration index (FICI) was determined according to the standard formula: FICI = (MIC of drug A in combination/MIC of drug A alone) + (MIC of drug B in combination/MIC of drug B alone).

Based on FICI values, drug interactions were classified as follows: synergistic (FICI ≤ 0.5), additive (0.5 < FICI ≤ 1.0), indifferent (1.0 < FICI ≤ 2.0), and antagonistic (FICI > 2.0).

#### 2.4.6 Stability assay

Following published procedures (Ko et al., 2020), we examined how physiological salt conditions affect Scymicrosin_7–26_’s efficacy. Bacterial strains (EC1, KP1, AB1, PA1, MRSA1) were grown overnight and adjusted to 1 × 10^6^ CFU/mL in MH broth. The peptide was serially diluted in MH broth supplemented with either (1) 4 μM FeCl₃, 2.5 mM CaCl₂, and 150 mM NaCl for salt stability assessment, or (2) 5, 10, and 20% (v/v) fetal bovine serum (FBS) for serum stability analysis. MIC determinations followed standard microdilution protocols, with triplicate measurements within each experiment and three separate experimental runs.

#### 2.4.7 Resistance induction assay

The potential for resistance development to Scymicrosin_7–26_ was investigated using a serial passage method (Yu et al., 2021). PA1 cultures were transferred to fresh MH medium supplemented with Scymicrosin_7–26_ at sub-MIC levels and cultivated at 37 °C. Cultures from 0.5 × MIC wells were harvested after 24 h, diluted 1:1000 in fresh medium, and exposed to a new gradient of peptide concentrations. This daily passaging was continued for 30 days. Polymyxin B and tigecycline were used as control antibiotics. With each transfer, the fold-increase in MIC compared to the original baseline was recorded.

#### 2.4.8 Biofilm formation inhibition assay

PA1 biofilm formation under Scymicrosin_7–26_ exposure was quantified in 96-well plates with peptide concentrations (0, 0.5, 1, 2, 4 × MIC). After 24 h static incubation (37 °C), wells were aspirated, phosphate-buffered saline (PBS)-washed, and methanol-fixed (10 min). Air-dried biofilms underwent crystal violet staining (1%, 20 min), distilled water washing, and ethanol elution for OD₅₉₅ measurement. Technical triplicates and three biological repeats were performed.

#### 2.4.9 Mature biofilm eradication assay

Mature PA1 biofilms were established in 96-well plates (24 h, 37 °C), washed with PBS, and challenged with Scymicrosin_7–26_ (0, 0.5, 1, 2, 4 × MIC) in fresh MH broth for 24 h at 37 °C. The remaining biofilm was then measured according to the crystal violet method in section 2.1.7.

#### 2.4.10 Cytotoxicity assay

The cytotoxicity of Scymicrosin_7–26_ was evaluated against RAW264.7, Beas-2B, HaCaT, and HEK293T cell lines. Following 24 h culture in complete medium (high-glucose DMEM with 10% FBS) at 1 × 10^4^ cells/well in 96-well plates, cells were treated with Scymicrosin_7–26_ (3–48 μM) in fresh medium. Blank controls (medium only) and negative controls (untreated cells) were established. Viability was determined after 24 h using CCK-8 assay (10 μL/well, 2 h incubation at 37 °C) with detection at 450 nm.

The cell survival rate was quantified by the formula:


$$\text{Cell Survival Rate}\;(\%)=({\mathrm{OD}}_{A}-{\mathrm{OD}}_{B})/({\mathrm{OD}}_{C}-{\mathrm{OD}}_{B})\times 100\%$$

The absorbance readings for the peptide-treated groups, blank control, and negative control were designated as OD_A_, OD_B_, and OD_C_, respectively. Six replicates were used for each condition.

#### 2.4.11 Hemolytic activity

Hemolysis assay was performed with 4% human erythrocyte suspensions. Erythrocytes were exposed to varying concentrations of Scymicrosin_7–26_, 1% Triton X-100 (positive control), and PBS (negative control) for 1 h at 37 °C. Following centrifugation at 4000 × g for 5 min (room temperature, RT), 100 μL of each supernatant was transferred to a 96-well plate. Hemoglobin release was determined by measuring absorbance at 540 nm.

The hemolysis rate was determined as follows:


$$\text{Hemolysis}\;(\%)=100\times [(A–A_{o})/(A_{T}–A_{o})]$$

In this formula, A, A₀, and A_T_ refer to the absorbance readings of the experimental groups, PBS control, and Triton X-100 control, respectively. All assays were performed in triplicate.

### 2.5 Elucidating the antimicrobial mechanism of Scymicrosin_7–26_

#### 2.5.1 Outer membrane permeability assay

N-phenyl-1-naphthylamine (NPN), a environment-sensitive fluorescent probe, was employed to examine the outer membrane disruption ability of Scymicrosin_7–26_. This probe shows minimal fluorescence in aqueous solution but markedly increased emission when incorporated into membrane hydrophobic compartments. In brief, PA1 suspensions (1 × 10^6^ CFU/mL) were loaded with 10 μM NPN for 10 min under dark conditions. After treatment with Scymicrosin_7–26_ at 1×, 2×, and 4 × MIC concentrations, along with polymyxin B control (4 μg/mL), fluorescence kinetics were monitored at 1-min intervals (excitation 350 nm, emission 420 nm) using a BioTek plate reader until signal equilibrium.

#### 2.5.2 Live/dead assay

The effect of Scymicrosin_7–26_ on bacterial membrane integrity was tested with the LIVE/DEAD® BacLight™ viability kit. PA1 and MRSA1 (1 × 10^6^ CFU/mL) treated with 1 × MIC peptide for 1 h were stained with SYTO 9/PI combination (20 μM and 20 μg/mL) for 15 min at 37 °C in darkness. Microscopic observation was conducted immediately using a fluorescence microscope.

#### 2.5.3 Scanning electron microscope (SEM)

The morphological effects of Scymicrosin_7–26_ on bacterial cells were investigated through SEM imaging, following a previously described method with slight modifications (Kalsy et al., 2020). PA1 and MRSA1 cultures in logarithmic growth phase were normalized to 1 × 10^8^ CFU/mL and exposed to Scymicrosin_7–26_ (1 × and 2 × MIC) for 1 h at 37 °C. After centrifugation (3,000 × g, 10 min) and triple PBS washing, cells were fixed in 2.5% glutaraldehyde (4 °C, overnight). Dehydration through graded ethanol series, critical point drying, and gold coating preceded SEM observation.

#### 2.5.4 Transmission electron microscope (TEM)

For TEM observation, samples were processed based on a previously described protocol with customized changes^35^. Bacterial pellets from peptide-treated cultures (prepared as in 2.2.3) underwent the following processing: primary fixation with 2.5% glutaraldehyde at 4 °C overnight; post-fixation with 1% osmium tetroxide for 2 h at 4 °C; rinsing with PBS; dehydration through a graded acetone series (50, 70, 90, and 100%); embedding in Epon 812 resin and thermal polymerization (70 °C, 48 h), 65–70 nm sections were prepared using a UC6 ultramicrotome. The sections were sequentially stained with 3% uranyl acetate and 1% lead citrate prior to TEM observation.

#### 2.5.5 DNA binding assay

The DNA-binding capability of Scymicrosin_7–26_ was analyzed by an agarose gel retardation assay, as previously reported (Xie et al., 2015). Genomic DNA isolation from PA1 and MRSA1 strains was performed with a commercial bacterial DNA extraction kit. Aliquots of DNA (approximately 400 ng in 10 μL TE buffer) were incubated with increasing concentrations of Scymicrosin_7–26_ (0, 3, 6, 12, 24, 48, and 96 μM) at room temperature for 30 min. After incubation, the reaction mixtures were analyzed by 1% agarose gel electrophoresis. A Bio-Rad gel imaging system was used to visualize DNA migration patterns.

#### 2.5.6 Bacterial reactive oxygen species (ROS) detection

Bacterial intracellular ROS production was monitored with the fluorescent indicator 2′,7′-dichlorodihydrofluorescein diacetate (DCFH-DA), following a published method (Jayathilaka et al., 2021). Bacterial suspensions (OD₆₀₀ ≈ 0.8) of PA1 and MRSA1 in PBS were loaded with 10 μg/mL DCFH-DA and continuously shaken at 37 °C for 1 h. Excess fluorescent probe was removed through three successive PBS washes. DCFH-DA-loaded bacteria were incubated with Scymicrosin_7–26_ gradient concentrations (0, 0.5, 1, 2, 4 × MIC) for 60 min. Species-appropriate positive controls (polymyxin B for PA1; lysostaphin for MRSA1) were included. Fluorescence intensity was measured with excitation at 485 nm and emission at 528 nm.

### 2.6 Anti-inflammatory effects and underlying mechanisms of scymicrosin_7–26_

#### 2.6.1 Modeling LPS-induced inflammation in RAW264.7 cells

RAW264.7 cells (1 × 10^6^ cells/mL) were distributed into 6-well plates (2 mL/well) containing high-glucose DMEM and incubated for 24 h at 37 °C with 5% CO₂. The study included five experimental conditions: (1) untreated control; (2) LPS-stimulated model (100 ng/mL); and (3–5) treatment groups receiving 2-h pretreatment with Scymicrosin_7–26_ (3, 6, or 12 μM) prior to 22-h LPS co-incubation. After treatment, culture media were harvested for subsequent analysis while adherent cells were processed for RNA and protein extraction.

#### 2.6.2 Reverse transcription quantitative polymerase chain reaction (RT-qPCR)

Cellular total RNA was isolated with Trizol reagent. Complementary DNA (cDNA) synthesis was carried out with the PrimeScript® Reverse Transcription Kit following the supplier’s instructions. Using PerfectStart® Green qPCR SuperMix, RT-qPCR analyses were carried out on a Thermo Fisher Applied Biosystems real-time PCR platform. All primer sequences utilized in this work are detailed in Supplementary Table S4.

#### 2.6.3 Enzyme-linked immunosorbent assay (ELISA)

Commercial ELISA kits were employed to evaluate the levels of IL-1β, IL-6, and TNF-*α* in cell culture supernatants, strictly adhering to the manufacturer’s protocols.

#### 2.6.4 Nitric oxide (NO) detection

According to the Griess reaction protocol in the Nitric Oxide Assay Kit, we measured nitrite accumulation in culture media as an indicator of nitric oxide production.

#### 2.6.5 Measurement of intracellular ROS in RAW264.7 cells

Following the respective treatment, cells underwent three PBS washes before being incubated with the fluorescent probe DCFH-DA (10 μM) for 30 min at 37 °C in darkness. After incubation with DCFH-DA, the cells were washed thoroughly with PBS to eliminate residual extracellular fluorophore. Intracellular ROS levels, indicated by fluorescence, were observed and imaged using a fluorescence microscope.

#### 2.6.6 Western blot analysis

Treated cells were subjected to cold PBS washes and RIPA buffer lysis on ice. Proteins were denatured (99 °C, 10 min) in loading buffer and electrophoresed on 10% SDS-polyacrylamide gels. Proteins were transferred to PVDF membranes, blocked with 5% BSA (1 h, RT). Primary antibody incubation (4 °C, overnight) preceded TBST washes and HRP-secondary antibody treatment (1 h, RT). Visualization used a chemiluminescent detector, with three biological replicates.

#### 2.6.7 LPS neutralization assay

The LPS neutralization activity of Scymicrosin_7–26_ was evaluated according to a previously described method (Nell et al., 2006). Briefly, the peptide at concentrations ranging from 1.5 to 48 μM was co-incubated with 100 ng/mL LPS at 37 °C for 30 min. Following incubation, residual LPS levels were measured using a commercial LPS detection kit according to the manufacturer’s instructions. The neutralization percentage was calculated based on the reduction in LPS activity relative to the control (without peptide).

#### 2.6.8 Cellular penetration assay

The membrane penetration capability of Scymicrosin_7–26_ was evaluated in RAW264.7 macrophages using inverted fluorescence microscopy. Cells were plated in 12-well plates at 5 × 10^5^ cells per well and adhered for 12 h. FITC-labeled peptide was administered in high-glucose DMEM at concentrations ranging from 0 to 12 μM for 1 h. Following treatment, cells were rinsed with PBS, fixed with 4% paraformaldehyde (20 min), and blocked with 10% goat serum. Immunostaining was performed using an anti-F4/80 primary antibody (1:500, overnight) followed by a Cy3-conjugated secondary antibody (1 h, room temperature). Nuclei were counterstained with DAPI after thorough washing. Fluorescence images were acquired using an inverted fluorescence microscope.

#### 2.6.9 Immunofluorescence staining

After overnight culture in 24-well plates (4 × 10^5^ cells/mL, 500 μL/well), RAW264.7 cells were treated, PBST-washed, and fixed with 4% PFA (15 min). Blocking with 5% BSA (1 h, RT) preceded anti-P65 primary antibody incubation (overnight, 4 °C). Cy3-conjugated secondary antibody was applied (1 h, RT, dark), followed by Hoechst 33342 nuclear staining (10 min) and fluorescence imaging.

### 2.7 Statistical analysis

GraphPad Prism 9 (GraphPad Software, CA, USA) was utilized for data analysis. Results are reported as mean ± SD. Comparative analyses included unpaired Student’s *t*-test for two groups and one-way ANOVA for multiple groups. Statistical significance was defined as *p < 0.05, **p < 0.01, ***p < 0.001, ****p < 0.0001.

## 3 Results

### 3.1 Efficacy and safety profile of Scymicrosin_7–26_ against multidrug-resistant bacteria

#### 3.1.1 Antimicrobial susceptibility testing

The antibacterial efficacy of Scymicrosin_7–26_ was initially assessed against a panel of five clinically prevalent multidrug-resistant bacteria isolated from respiratory specimens. The peptide demonstrated antibacterial effects across all tested strains. Analysis of MIC₅₀ and MIC₉₀ values revealed that Scymicrosin_7–26_ was most active against *Escherichia coli*, *Acinetobacter baumannii*, and methicillin-resistant *Staphylococcus aureus* (MRSA), followed by *Klebsiella pneumoniae*, with *Pseudomonas aeruginosa* exhibiting the highest MIC values (Table 1). The susceptibility of the five strains selected according to the aforementioned criteria to Scymicrosin_7–26_ is summarized in Table 2.

**Table 1:** Antimicrobial activities of scymicrosin_7–26._

Clinical strains	Sample size (*n*)	MIC and the number of strains							MIC_50_ (μM)	MIC_90_ (μM)	GMIC (μM)	95% CI (μM)
		<3	3	6	12	24	48	>48				
MDR *Escherichia coli*	46	0	20	24	2	0	0	0	6	6	4.57	4.06–5.15
MDR *P.aeruginosa*	22	0	0	2	5	11	3	1	24	48	20.5	15.7–26.8
MDR *K.pneumoniae*	23	0	0	16	7	0	0	0	6	12	7.41	6.43–8.54
MDR *A. baumannii*	28	3	15	10	0	0	0	0	3	6	3.98	3.48–4.55
MRSA	18	0	8	10	0	0	0	0	6	6	4.41	3.70–5.26

MIC: Minimum Inhibitory Concentration; MIC_50_: Minimum Inhibitory Concentration for 50% of the isolates; MIC_90_: Minimum Inhibitory Concentration for 90% of the isolates. GMIC: Geometric Mean Minimum Inhibitory Concentration. 95% CI: The 95% Confidence Interval.

**Table 2:** MIC and MBC of Scymicrosin_7–26._ against the five experimental bacterial strains.

Strains	‌No.‌	MIC (μM)	MBC (μM)
EC1	208,815	6	12
PA1	110,809	24	96
KP1	302,404	6	12
AB1	116,657	3	6
MRSA1	218,335	6	6

MBC, minimum bactericidal concentration.

#### 3.1.2 Growth kinetics analysis

**Figure 1:**
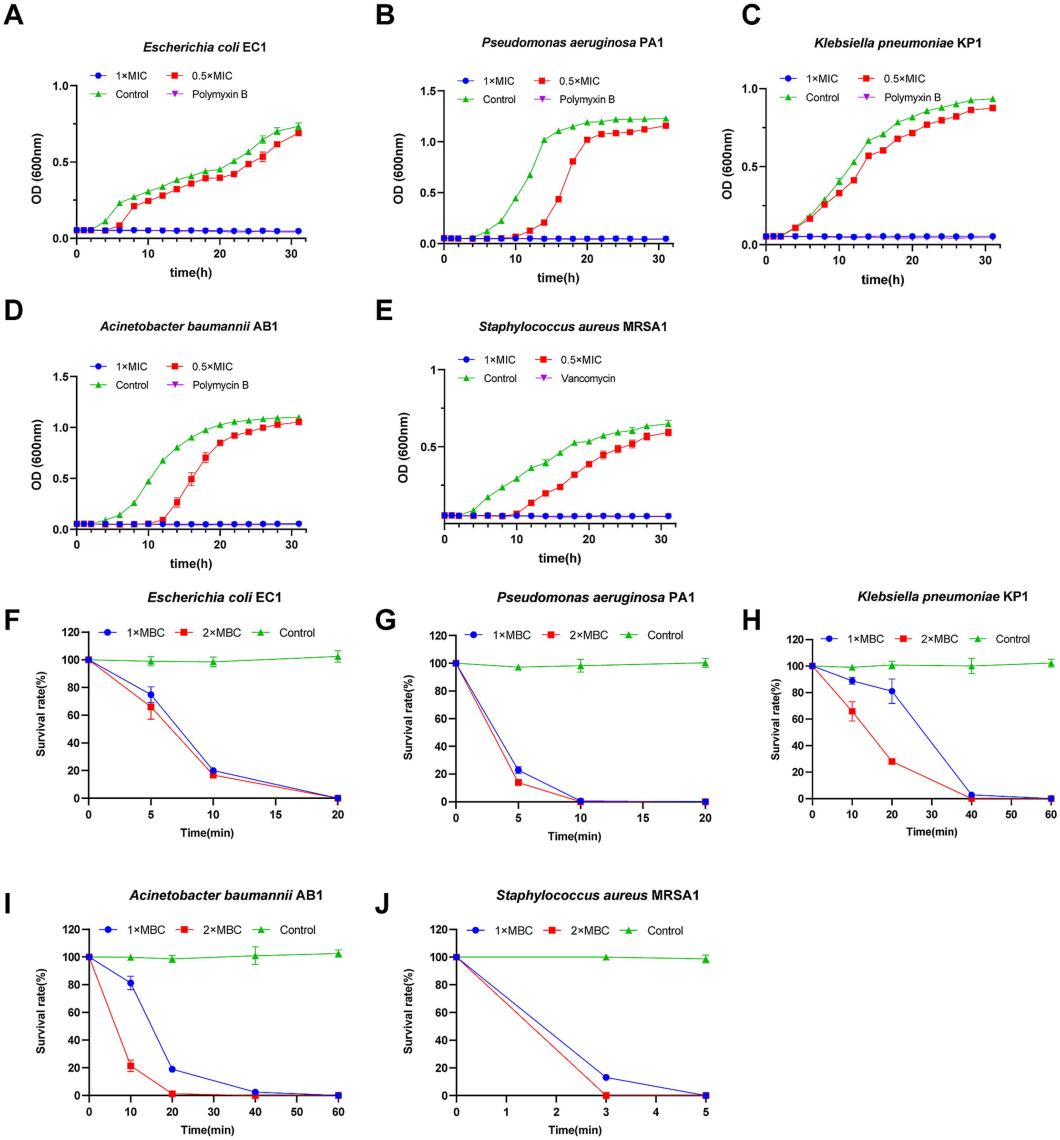
Effects of Scymicrosin_7–26_ on the growth and viability of multidrug-resistant bacteria. **(A–E)** Growth kinetics of five multidrug-resistant clinical isolates from respiratory specimens under treatment with Scymicrosin_7–26_. **(A)** *Escherichia coli* EC1, 1 × MIC = 6 μM. **(B)** *Pseudomonas aeruginosa* PA1, 1 × MIC = 24 μM. **(C)** *Klebsiella pneumoniae* KP1, 1 × MIC = 6 μM. **(D)** *Acinetobacter baumannii* AB1, 1 × MIC = 3 μM. **(E)** *Staphylococcus aureus* MRSA1, 1 × MIC = 6 μM. **(F–J)** Time-kill kinetics of Scymicrosin_7–26_ against the five multidrug-resistant clinical isolates. **(F)** *Escherichia coli* EC1, 1 × MBC = 12 μM. **(G)** *Pseudomonas aeruginosa* PA1, 1 × MBC = 96 μM. **(H)** *Klebsiella pneumoniae* KP1, 1 × MBC = 12 μM. **(I)** *Acinetobacter baumannii* AB1, 1 × MBC = 6 μM. **(J)** *Staphylococcus aureus* MRSA1, 1 × MBC = 6 μM. Positive control groups: Polymyxin B was used at 1 × MIC = 4 μg/mL for EC1, PA1, KP1, and AB1; vancomycin was used at 1 × MIC = 2 μg/mL for MRSA1. The negative control groups were treated with broth without Scymicrosin_7–26_.

Figures 1A–E illustrates the growth kinetics of the tested bacterial strains. Sub-inhibitory concentrations (0.5 × MIC) of Scymicrosin_7–26_ significantly retarded the growth of all five strains. At the 1 × MIC concentration, bacterial growth was completely suppressed. Similar inhibitory profiles were documented in positive control groups administered 1 × MIC of either polymyxin B or vancomycin. These findings indicate that Scymicrosin_7–26_ exerts concentration-dependent suppression of bacterial growth in the tested multidrug-resistant pathogens.

#### 3.1.3 Time-kill kinetics

Time-kill assays were performed to dynamically monitor the bactericidal activity of Scymicrosin_7–26_. As shown in Figures 1F–J, exposure to 1 × MBC of the peptide resulted in complete eradication of strains KP1 and AB1 within 60 min, EC1 and PA1 within 20 min, and MRSA1 within 5 min. When the concentration was increased to 2 × MBC, the killing kinetics were accelerated: KP1 and AB1 were eliminated within 40 min, EC1 within 20 min, PA1 within 10 min, and MRSA1 within 3 min. Notably, the killing rate for EC1 was more rapid at 2 × MBC during the initial 20-min period compared to 1 × MBC.

#### 3.1.4 Checkerboard assay

Combination therapy was assessed using the checkerboard microdilution method. As summarized in Supplementary Table S5, the combination of Scymicrosin_7–26_ with polymyxin B, tigecycline, imipenem, amikacin, vancomycin, or lincomycin against strains AB1, KP1, EC1, PA1, and MR1 yielded fractional inhibitory concentration index (FICI) values all below 2.00, indicating no antagonistic interactions were observed for any of the tested combinations.

#### 3.1.5 Stability assay

The stability of Scymicrosin_7–26_’s antibacterial activity under a range of physiological ion and fetal bovine serum (FBS) concentrations was evaluated. In the presence of a physiological Na^+^ concentration, only a marginal increase in MIC was noted for strains EC1, KP1, and MRSA1. Exposure to a physiological concentration of Ca^2+^ resulted in a modest increase in the MIC for all five bacterial strains, with the effect being most pronounced for PA1. Conversely, physiological Fe^3+^ concentration did not alter the MIC against any of the strains. These results demonstrate that Scymicrosin_7–26_ retains robust antibacterial activity in environments mimicking physiological salt conditions. The MIC values remained unchanged in the presence of 5% fetal bovine serum (FBS). However, they increased at higher FBS concentrations (10 and 20%). This effect was most pronounced in strain PA1, while the other tested strains showed only moderate changes (Table 3).

**Table 3:** *In vitro* stability of Scymicrosin_7–26_ in salt ions and fetal bovine serum (FBS).

Strains ‌no.‌		MIC (μM)						
		Control	NaCl	CaCl_2_	FeCl_3_	5% FBS	10% FBS	20% FBS
EC1	218,335	6	12	48	6	6	12	24
PA1	116,657	24	24	>192	24	24	96	>192
KP1	302,404	6	24	12	6	6	12	24
AB1	110,809	3	3	24	3	3	3	24
MRSA1	208,815	6	12	24	6	6	12	24

#### 3.1.6 Resistance induction

**Figure 2:**
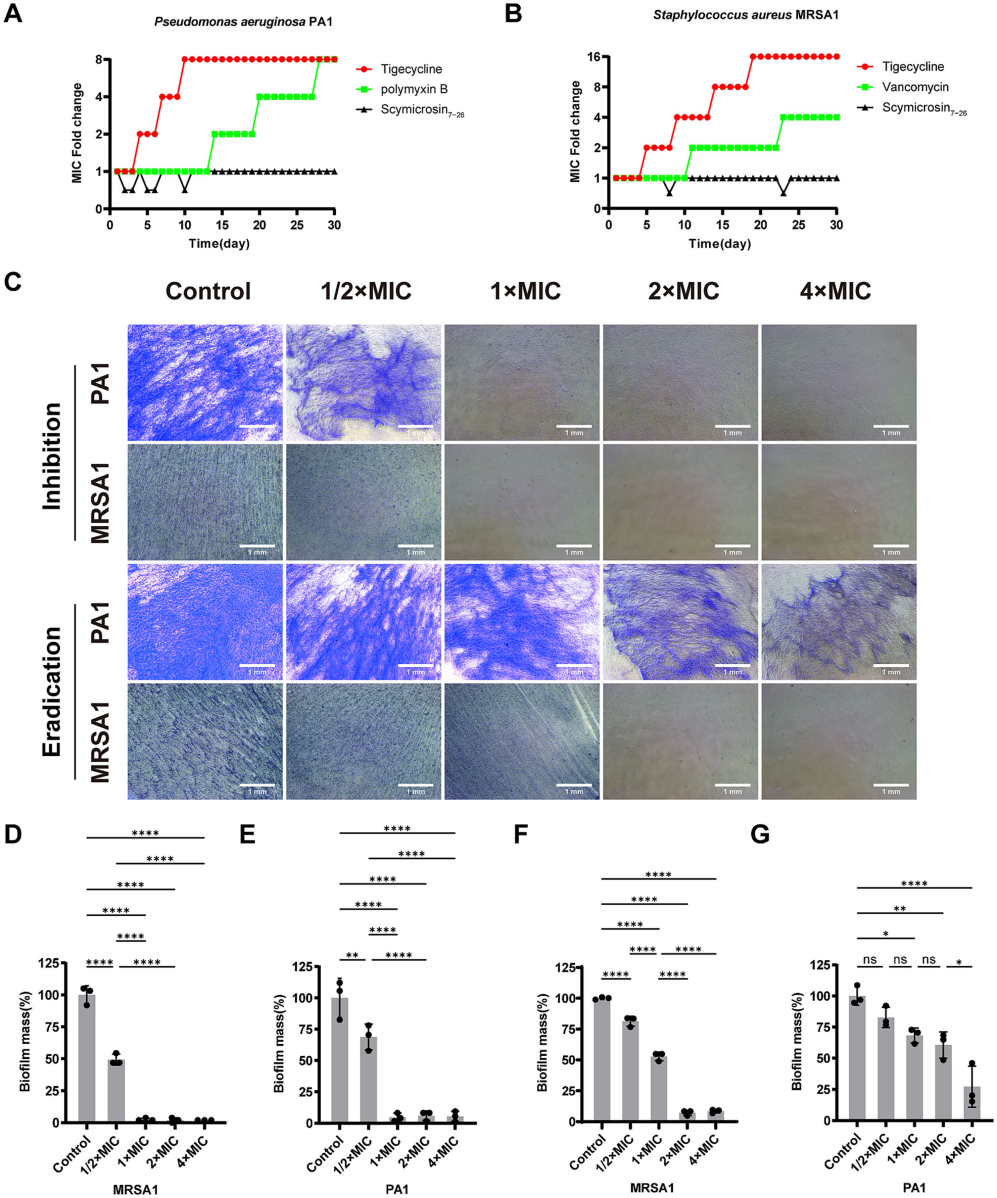
Effects of Scymicrosin_7–26_ on bacterial resistance and biofilms. **(A)** Fold changes in the MIC of strain PA1 after 30 days of serial passaging in the presence of Scymicrosin_7–26_, tigecycline, or polymyxin B. **(B)** Fold changes in the MIC of strain MRSA1 following 30 days of serial exposure to Scymicrosin_7–26_, tigecycline, or vancomycin. **(C)** Inhibitory effects of Scymicrosin_7–26_ on nascent biofilm formation and eradication of preformed mature biofilms in PA1 and MRSA1. Blue areas indicate crystal violet-stained biofilm. For PA1, 1 × MIC = 24 μM; for MRSA1, 1 × MIC = 6 μM. Scale bars: 1 mm. **(D)** Quantification of Scymicrosin_7–26_–mediated inhibition of nascent MRSA1 biofilm formation. **(E)** Quantification of Scymicrosin_7–26_–mediated inhibition of nascent PA1 biofilm formation. **(F)** Quantification of Scymicrosin_7–26_–mediated eradication of mature MRSA1 biofilm. **(G)** Quantification of Scymicrosin_7–26_–mediated eradication of mature PA1 biofilm. Data are presented as mean ± SD (*n* = 3). *p < 0.05, **p < 0.01, ****p < 0.0001.

A serial passage experiment was conducted to assess the potential for resistance development. After 30 days of continuous exposure, the MIC of tigecycline and polymyxin B against PA1 increased by 8-fold compared to the baseline, whereas the MIC of Scymicrosin_7–26_ remained unchanged (Figure 2A). For MRSA1, the MIC of tigecycline and vancomycin increased by 16-fold and 4-fold, respectively, while the MIC of Scymicrosin_7–26_ again showed no increase (Figure 2B).

#### 3.1.7 Biofilm inhibition and eradication

Scymicrosin_7–26_ effectively inhibited biofilm formation at concentrations as low as 0.5 × MIC for both PA1 and MRSA1, with complete inhibition achieved at higher concentrations. Against pre-formed mature biofilms, the peptide also displayed eradication activity. At 0.5 × MIC, a partial removal effect was observed. For PA1, the biofilm biomass was reduced to 26.48% of the control at 4 × MIC. For MRSA1, the biomass decreased to 52.83% at 1 × MIC and was nearly completely eradicated at concentrations ≥2 × MIC (Figures 2C–G).

#### 3.1.8 Cytotoxicity assessment

**Figure 3:**
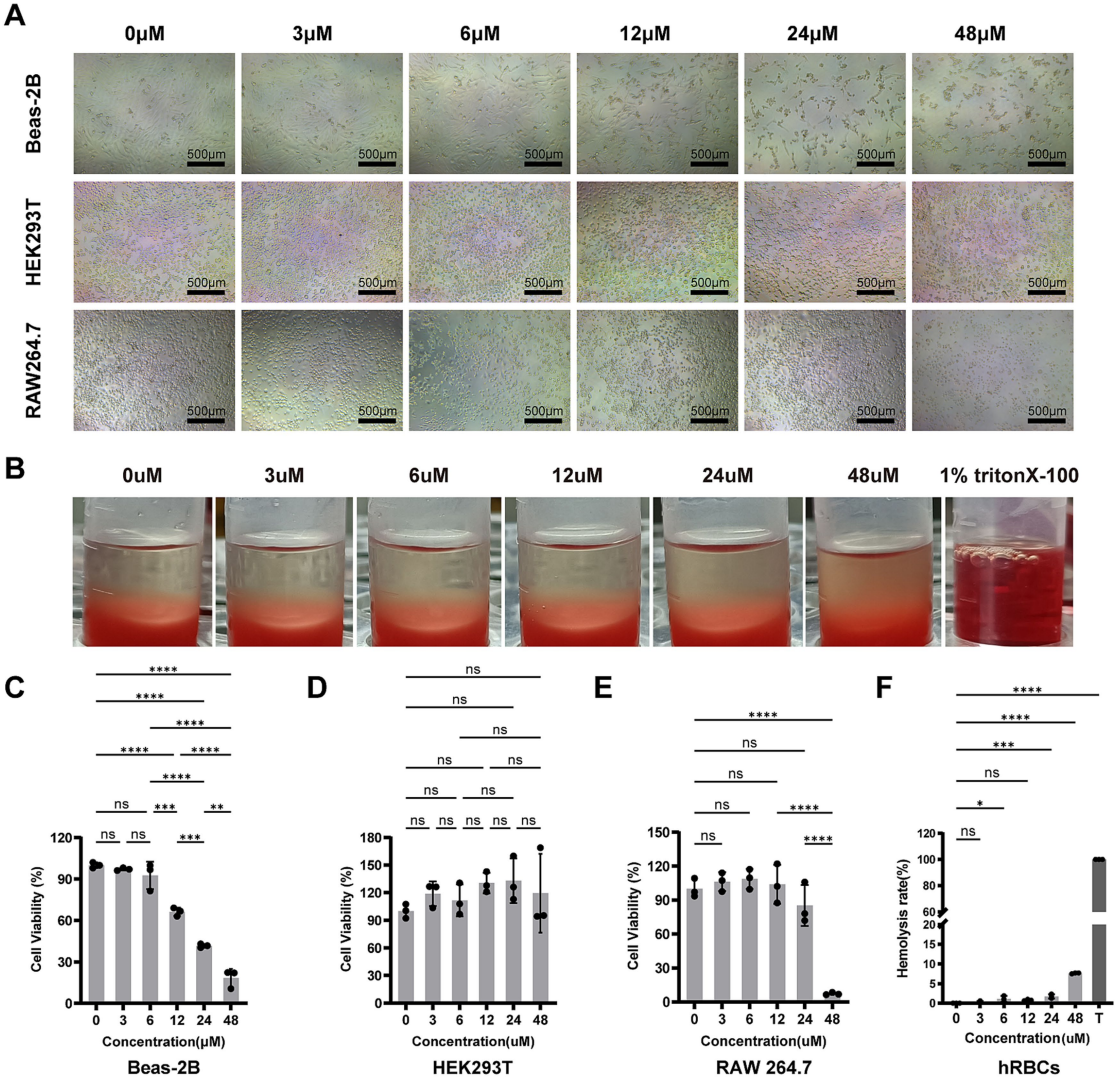
Cytotoxicity and hemolytic activity of Scymicrosin_7–26_. **(A)** Morphological changes in three cell lines (Beas-2B, HEK293T, and RAW264.7) after treatment with varying concentrations of Scymicrosin_7–26_. Scale bars: 500 μm. **(B)** Hemolytic activity of Scymicrosin_7–26_ against human red blood cells at different concentrations. T represents 1% Triton X-100 (positive control). **(C–E)** Cytotoxicity of Scymicrosin_7–26_ toward Beas-2B, HEK293T, and RAW264.7 cells, assessed by CCK-8 assay. **(F)** Hemolysis rate of human red blood cells treated with Scymicrosin_7–26_. Data are presented as mean ± SD (*n* = 3). *p < 0.05, **p < 0.01, ***p < 0.001, ****p < 0.0001.

The cytotoxicity profile of Scymicrosin_7–26_ was assessed in Beas-2B, HEK293T, and RAW264.7 cell lines. As shown in Figures 3C–E, the peptide exhibited low to negligible cytotoxicity across a range of concentrations. Cell morphology remained normal at non-cytotoxic concentrations, whereas characteristic shrinkage and fragmentation were observed at cytotoxic doses (Figure 3A).

#### 3.1.9 Hemolytic activity

The hemolysis rate was calculated based on the OD_540_ value of the supernatant. As illustrated in Figures 3B,F, the peptide induced no significant hemolysis at concentrations up to 12 μM. Even at 24 μM and 48 μM, the hemolysis rates remained very low, at 1.76 and 7.63%, respectively, indicating a high hemocompatibility within its effective antibacterial concentration range.

### 3.2 Elucidating the antimicrobial mechanism of Scymicrosin_7–26_

#### 3.2.1 Outer membrane permeabilization

**Figure 4:**
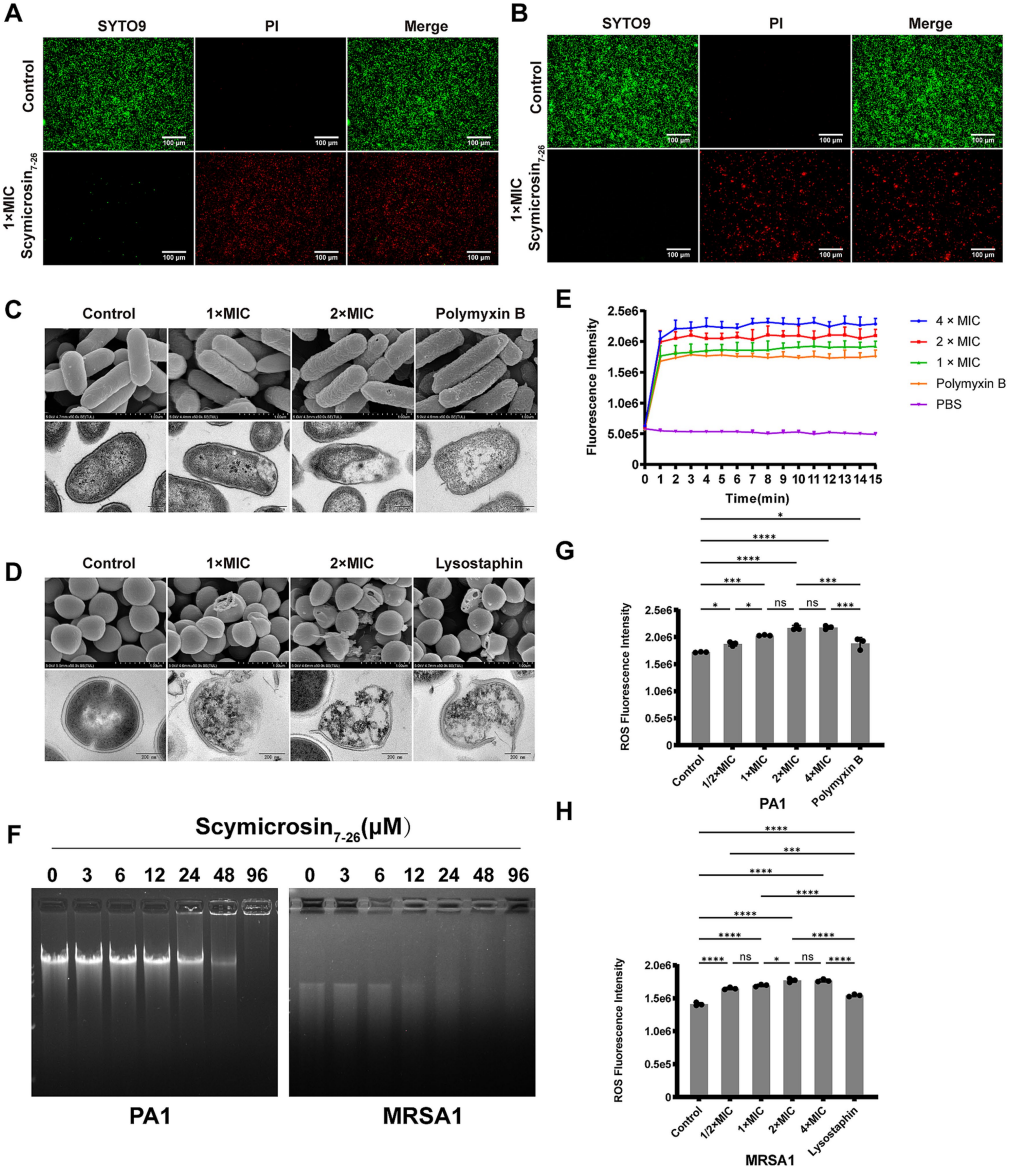
Antibacterial mechanism of Scymicrosin_7–26_ against multidrug-resistant bacteria *in vitro***. (A)** Live/dead fluorescence staining of *Pseudomonas aeruginosa* PA1 untreated (control) or treated with 1 × MIC (24 μM) Scymicrosin_7–26_. Red and green fluorescence indicate dead and live bacteria, respectively. Scale bars: 100 μm. **(B)** Live/dead staining of *Staphylococcus aureus* MRSA1 untreated or treated with 1 × MIC (6 μM) Scymicrosin_7–26_. Scale bars: 100 μm. **(C, D)** Scanning and transmission electron micrographs showing structural damage to PA1 and MRSA1 after treatment with Scymicrosin_7–26_ at 0×, 1×, and 2 × MIC. Polymyxin B and lysostaphin were used as positive controls. Scale bars: 1 μm (SEM) and 200 nm (TEM). **(E)** Continuous fluorescence monitoring of outer membrane permeability in PA1 after Scymicrosin_7–26_ treatment. **(F)** Agarose gel retardation assay showing binding of Scymicrosin_7–26_ to genomic DNA of PA1 and MRSA1. **(G, H)** Intracellular ROS levels in PA1 and MRSA1 after treatment with Scymicrosin_7–26_ at different concentrations. Results are presented as mean ± SD (*n* = 3). ^*^p < 0.05, ***p < 0.001, ****p < 0.0001.

As shown in Figure 4E, fluorescence intensity in all groups reached its peak within 2 min. Notably, all Scymicrosin_7–26_ treatment groups exhibited higher fluorescence intensities than the polymyxin B control group, with a clear concentration-dependent increase. These results indicate that Scymicrosin_7–26_ can quickly and effectively permeabilizes the outer membrane of *P. aeruginosa* PA1.

#### 3.2.2 Membrane integrity assessment

As demonstrated in Figure 4A, treatment with 1 × MIC Scymicrosin_7–26_ resulted in nearly complete red fluorescence (indicating dead cells) in PA1 cultures, confirming severe membrane damage. In contrast, untreated control cells exhibited predominantly green fluorescence (viable cells), indicating intact membranes. A similar pattern was observed for MRSA1 (Figure 4B), where exposure to 1 × MIC Scymicrosin_7–26_ also induced extensive membrane disruption, as evidenced by the dominance of red fluorescence.

#### 3.2.3 Scanning electron microscope (SEM)

Figures 4C,D illustrates the morphological changes in the representative Gram-negative strain PA1 and Gram-positive strain MRSA1. Untreated control cells displayed smooth, intact surfaces. Following treatment with 1 × MIC Scymicrosin_7–26_, PA1 cells exhibited widespread surface wrinkling, while MRSA1 cells showed visible deformation and damage. These morphological disruptions were more severe at 2 × MIC. Interestingly, the surface damage pattern induced by Scymicrosin_7–26_ differed from that caused by polymyxin B (which induced vesicle formation on PA1), suggesting a distinct mechanism of action for the antimicrobial peptide.

#### 3.2.4 Transmission electron microscope (TEM)

Ultrastructural changes were further investigated by TEM (Figures 4C,D). Control cells of both strains exhibited intact membranes, dense cytoplasm, and no content leakage. After 1-h exposure to 1 × MIC Scymicrosin_7–26_, PA1 bacterial cells displayed visible dissociation of the inner membrane from the cell wall and partial cytoplasmic leakage. At 2 × MIC, cell boundaries became blurred, surface structures were severely compromised, and content leakage was exacerbated. Polymyxin B treatment resulted in cytoplasmic loosening in PA1. For MRSA1, 1 × MIC Scymicrosin_7–26_ induced substantial cell lysis and content release, which intensified at 2 × MIC, resembling the effects observed with lysostaphin treatment. These TEM observations corroborate the SEM findings, confirming the membrane-disruptive action of Scymicrosin_7–26_.

#### 3.2.5 DNA binding affinity

To investigate possible intracellular mechanisms, we examined the DNA-binding affinity of Scymicrosin_7–26_. As shown in Figure 4F, Scymicrosin_7–26_ began to retard the migration of PA1 genomic DNA at 24 μM, while MRSA1 DNA showed retardation at 12 μM. The retardation effect intensified with increasing peptide concentrations, suggesting that Scymicrosin_7–26_ may contribute to bacterial cell death by binding to genomic DNA.

#### 3.2.6 Bacterial reactive oxygen species (ROS) generation

Reactive oxygen species (ROS) are oxidative molecules produced under cellular stress, which are implicated in cellular damage and can ultimately induce cell death. As shown in Figures 4G,H, treatment with a sub-inhibitory concentration (0.5 × MIC) of Scymicrosin_7–26_ already elevated intracellular ROS levels in both PA1 and MRSA1. Dose-responsive ROS generation was detected in both strains at higher peptide concentrations, with ROS levels surpassing those induced by polymyxin B or lysostaphin treatments.

### 3.3 The anti-inflammatory effect of Scymicrosin_7–26_

#### 3.3.1 Cytotoxicity assessment in RAW 264.7 cells

**Figure 5:**
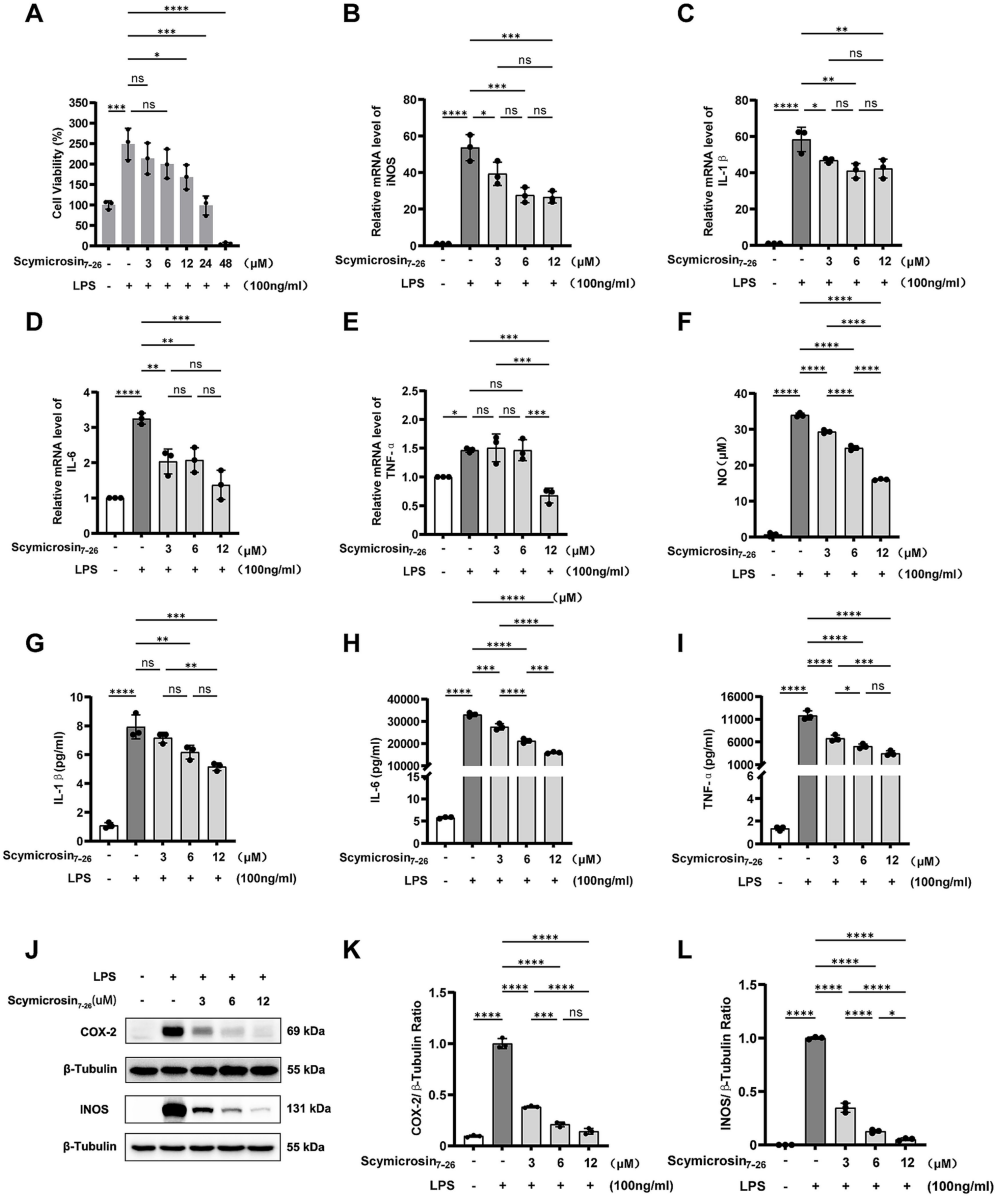
Effect of Scymicrosin_7–26_ on LPS-induced inflammatory response in RAW264.7 cells. **(A)** Cytotoxicity of LPS and/or Scymicrosin_7–26_ assessed by CCK-8 assay. **(B–E)** RT-qPCR analysis of *iNOS*, *IL-1β*, *IL-6*, and *TNF-α* mRNA expression levels. **(F)** Nitric oxide (NO) production measured by Griess assay. **(G–I)** ELISA quantification of IL-1β, IL-6, and TNF-α levels in cell culture supernatants. **(J–L)** Western blot analysis of COX-2 and *iNOS* protein expression. Results are presented as mean ± SD (*n* = 3). *p < 0.05, **p < 0.01, ***p < 0.001, ****p < 0.0001.

RAW264.7 cell viability was remarkably enhanced by LPS stimulation, reaching 249% of control values (Figure 5A). Scymicrosin_7–26_ administration at 3–24 μM concentrations produced a dose-responsive reduction in cellular viability, normalizing it to baseline levels. This observed reduction in cell viability is not a result of cytotoxicity, but rather stems from the inhibition of LPS-induced proliferative signaling, an effect potentially associated with activation of the Akt pathway (Gao et al., 2022). Exposure to 48 μM of the peptide drastically suppressed cell survival to 5.8%. Consequently, 3, 6, and 12 μM doses were chosen for follow-up anti-inflammatory experiments.

#### 3.3.2 mRNA expression of inflammatory mediators

Transcript expression of pivotal inflammatory mediators—IL-1β, IL-6, TNF-*α*, and inducible nitric oxide (iNOS) was measured by RT-qPCR. According to Figure 5B-E, LPS challenge significantly enhanced the transcriptional activity of all four investigated genes. However, treatment with Scymicrosin_7–26_ resulted in a concentration-dependent suppression of their mRNA expression. These data suggest that the peptide effectively inhibits the transcription of inflammatory mediators in the established RAW 264.7 inflammation model.

#### 3.3.3 Secretion of TNF-α, IL-1β, IL-6, and NO

The Griess assay and ELISA were employed to determine the levels of NO and cytokine concentrations (IL-1β, IL-6, TNF-α) in culture supernatants, respectively. Under LPS stimulation, all four inflammatory markers were significantly elevated (Figures 5F–I). Treatment with Scymicrosin_7–26_ led to a notable reduction in the production of IL-1β, IL-6, TNF-α and NO, indicating that Scymicrosin_7–26_ can effectively attenuate the release of key inflammatory factors in this cellular model.

#### 3.3.4 Protein expression levels of iNOS and COX-2

The anti-inflammatory effects of Scymicrosin_7–26_ were further substantiated through examination of iNOS and COX-2 protein expression. As depicted in Figure 5J–L, LPS challenge markedly upregulated both iNOS and COX-2 protein levels, whereas Scymicrosin_7–26_ treatment produced a concentration-dependent suppression of their expression. These results demonstrate that the peptide also inhibits the expression of intracellular inflammatory enzymes in macrophages, further supporting its role in modulating inflammatory signaling.

### 3.4 Elucidating the anti-inflammatory mechanism of Scymicrosin_7–26_

#### 3.4.1 LPS neutralizing activity

**Figure 6:**
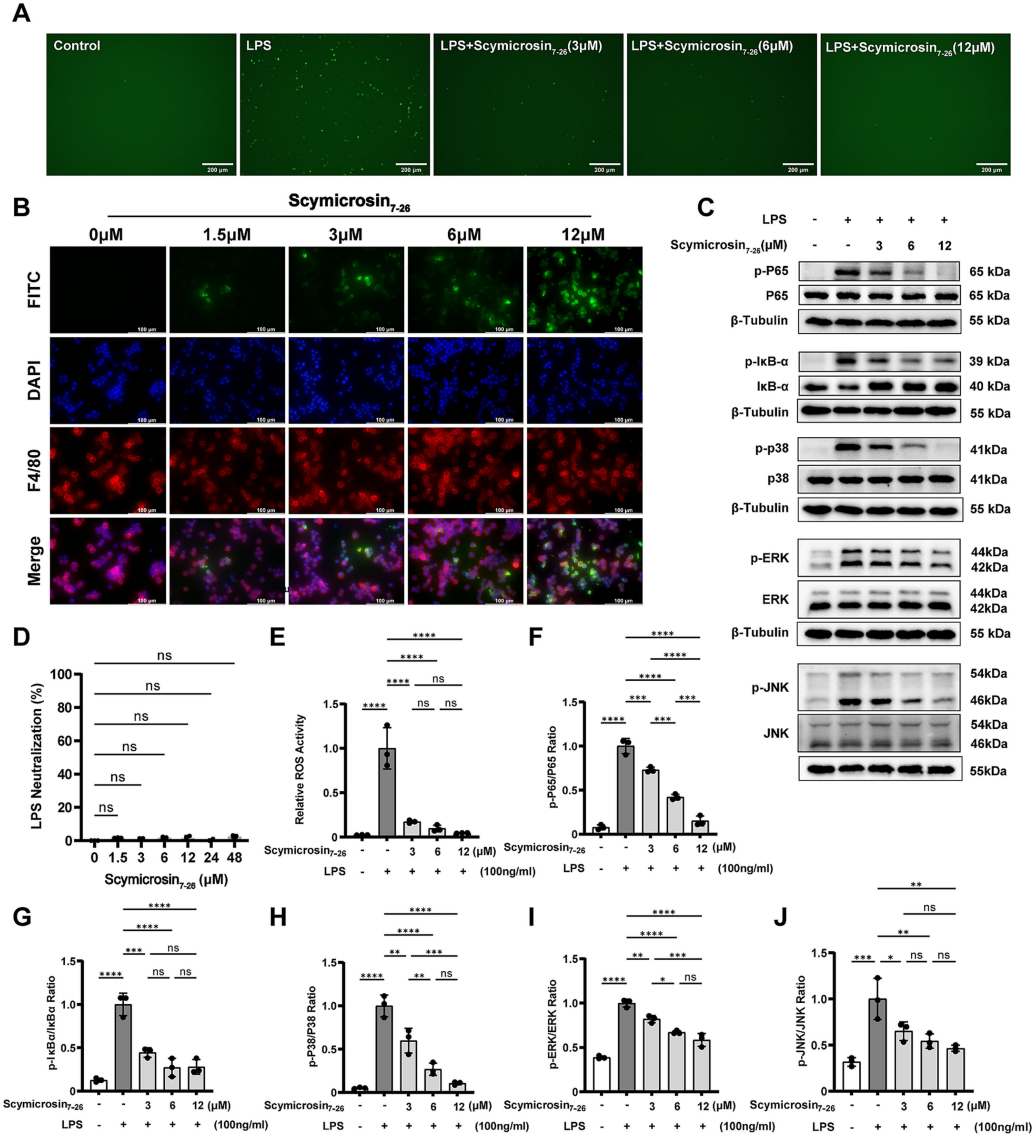
Mechanism of the anti-inflammatory effect of Scymicrosin_7–26_ *in vitro*. **(A, E)** Intracellular ROS levels in LPS-stimulated RAW264.7 macrophages treated with Scymicrosin_7–26_. Scale bars: 200 μm. **(D)** Neutralization rate of Scymicrosin_7–26_ against LPS. **(B)** Internalization of Scymicrosin_7–26_ into RAW264.7 cells. Scale bars: 100 μm. **(C,F–J)** Effects of Scymicrosin_7–26_ on the NF-κB and MAPK signaling pathways in RAW264.7 cells. ^*^p < 0.05, ^**^p < 0.01, ^***^p < 0.001, ^****^p < 0.0001 *vs.* control group.

In the LPS neutralization assay, incubation of LPS with Scymicrosin_7–26_ across a concentration range of 1.5–48 μM demonstrated no significant difference in neutralization rate compared to the 0 μM control group (Figure 6D). This indicates that Scymicrosin_7–26_, at these concentrations, does not neutralize LPS under the applied *in vitro* conditions.

#### 3.4.2 Cell-penetrating activity

RAW264.7 cells were stained with the macrophage surface marker F4/80 for the plasma membrane, DAPI for nuclei, and FITC-labeled Scymicrosin_7–26_ for the peptide localization. As shown in Figure 6B, no FITC green fluorescence was observed in the control group without Scymicrosin_7–26_. At 1.5 μM, faint fluorescent signals began to appear on the plasma membrane and within the cytoplasm of a small number of RAW264.7 cells. With increasing peptide concentrations, both the intensity and distribution of green fluorescence intensified in a dose-dependent manner, showing clear localization to cellular membranes and cytoplasmic regions. These results demonstrate that Scymicrosin_7–26_ effectively enters RAW264.7 cells in a concentration-dependent manner within the tested range of 1.5–12 μM.

#### 3.4.3 Attenuation of intracellular ROS in LPS-stimulated macrophages

When stimulated, immune cells produce diverse ROS that not only cause tissue damage but also perpetuate inflammatory cascades (Chen et al., 2020). As shown in Figure 6A, Scymicrosin_7–26_ treatment effectively reduced ROS generation in LPS-activated RAW 264.7 cells (Figure 6E).

#### 3.4.4 Modulation of the MAPK signaling pathway

The MAPK pathway represents a central regulator of inflammation, with its core components—P38, ERK, and JNK—playing critical roles (Haftcheshmeh et al., 2022). The results revealed that LPS treatment significantly upregulated the phosphorylation levels of P38, ERK, and JNK (Figure 6C). Quantitative analysis further confirmed these observations, showing significant elevations in the p-P38/P38, p-ERK/ERK, and p-JNK/JNK ratios (Figures 6H–J). Treatment with Scymicrosin_7–26_ concentration-dependently reversed these phosphorylation events, indicating that the peptide exerts its anti-inflammatory effects through suppression of MAPK pathway activation.

#### 3.4.5 Suppression of the NF-κB signaling pathway

The NF-κB signaling pathway serves as another critical regulator of inflammation. Upon LPS stimulation, activation of this pathway promotes the onset of inflammatory responses (Haftcheshmeh et al., 2022). To investigate whether Scymicrosin_7–26_ modulates the NF-κB pathway, we examined key phosphorylation events in this signaling cascade. LPS stimulation significantly enhanced the phosphorylation of both p65 and IκBα (Figures 6C,F,G). While total p65 levels remained consistent across groups, Scymicrosin_7–26_ treatment progressively reduced phospho-p65 and phospho-IκBα levels in a dose-dependent manner. Additionally, the LPS-induced degradation of IκBα was effectively counteracted by peptide intervention. These collective findings demonstrate that Scymicrosin_7–26_ inhibits NF-κB pathway activation in macrophages.

#### 3.4.6 Inhibition of NF-κB p65 nuclear translocation

**Figure 7:**
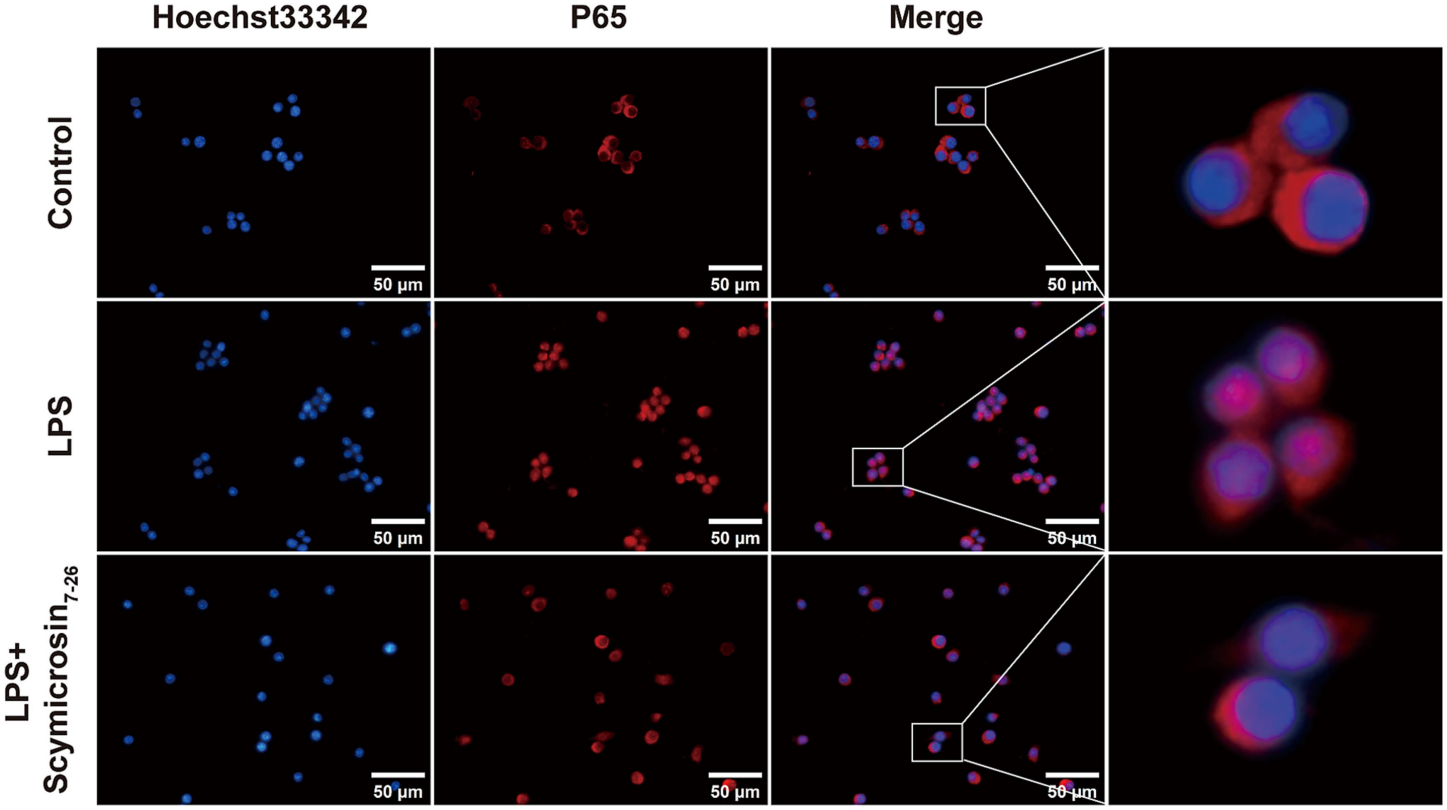
Effect of Scymicrosin_7–26_ on NF-κB p65 nuclear translocation. Scale bars: 50 μm.

Under stimulation by LPS or other pro-inflammatory factors, the cytoplasmic nuclear factor kappa B subunit p65 (p65) undergoes phosphorylation, transitioning from its non-phosphorylated state. The phosphorylated p65 (p-p65) subsequently translocates into the nucleus, where it binds to specific target genes and regulates their transcriptional expression, thereby modulating the expression of inflammatory mediators and other physiological responses (Florio et al., 2022). Immunofluorescence analysis (Figure 7) revealed minimal nuclear p65 signal in control cells. LPS stimulation induced pronounced p65 nuclear accumulation, whereas Scymicrosin_7–26_ treatment significantly reduced p65 nuclear translocation. The nuclear translocation of p65 is a central event in NF-κB pathway activation. The results establish that Scymicrosin_7–26_ mediates its anti-inflammatory activity by attenuating NF-κB signal transduction.

## 4 Discussion

The escalating prevalence of antimicrobial resistance is primarily driven by the widespread overuse of antibiotics. Over recent decades, excessive antibiotic usage has accelerated the emergence and dissemination of multidrug-resistant (MDR) bacterial strains, progressively undermining the efficacy of conventional antibiotics in infection management. Confronted by the growing threat of MDR pathogens to human and animal health, there is an urgent need to strengthen antibiotic stewardship while actively developing novel antimicrobial agents (Salama et al., 2021; Zhong et al., 2024; Li et al., 2025). Antimicrobial peptides (AMPs) derived from aquatic organisms—including fish, crustaceans, mollusks, and algae—have attracted considerable interest as promising therapeutic candidates. In this study, we demonstrated that Scymicrosin_7–26_, an AMP identified from *Scylla paramamosain*, exhibits rapid and broad-spectrum antibacterial activity against five types of clinically isolated MDR bacterial strains (Figure 1). As summarized in Supplementary Table S3, this study expands upon previous research on antimicrobial peptides such as AR-23 and Melectin by testing against an extended panel of clinical multidrug-resistant isolates. Under the limited testing conditions, the absence of antagonism between Scymicrosin_7–26_ and co-administered antibiotics enhances its potential as a viable candidate for combination therapy (Supplementary Table S5).

Cationic antimicrobial peptides typically initiate antibacterial action through electrostatic interactions with negatively charged bacterial membranes. The presence of physiological cations such as Na^+^, Ca^2+^, and Fe^3+^ can compete with these interactions, often leading to reduced peptide activity under high-salt conditions (Zhu et al., 2014). Our stability assays, conducted under physiologically relevant ion concentrations, revealed that Scymicrosin_7–26_ maintained nearly full antibacterial potency in the presence of Na^+^ and Fe^3+^. Although a moderate reduction in activity was observed in Ca^2+^-supplemented medium, a degree of antibacterial activity was preserved in the peptide. (Table 3). For systemic use, AMPs must remain stable in blood. Since serum proteases like trypsin rapidly degrade natural AMPs (Santos-Filho et al., 2017), we assessed Scymicrosin_7–26_’s stability in fetal bovine serum. Although activity declined faster in serum compared to ionic conditions, residual antibacterial activity was observed.

When subjected to continuous exposure to antimicrobial agents, bacteria may develop resistance through various molecular mechanisms (Habteweld and Asfaw, 2023). However, serial passaging experiments indicated no detectable resistance development in *Pseudomonas aeruginosa* or MRSA after prolonged exposure to Scymicrosin_7–26_. Multidrug-resistant strains commonly exhibit an enhanced capacity for biofilm formation. This mode of growth significantly increases their tolerance to both host immune defenses and antimicrobial agents. Consequently, biofilm-associated infections are notoriously difficult to eradicate and represent a leading cause of persistent and fatal infections (Senobar Tahaei et al., 2021; Zhou et al., 2023). Our data show that Scymicrosin_7–26_ can inhibits biofilm formation and disrupts mature biofilms (Figure 2).

In addition to antibacterial potency, biosafety is a critical determinant for the clinical translation of AMPs. The cytotoxicity and hemolytic activity of antimicrobial peptides are closely linked to their structural characteristics, such as hydrophobicity, net charge, and chemical modifications. Although many conventional antimicrobial peptides, such as SAAP-148 and AR-23, exhibit potent antimicrobial efficacy, their high cytotoxicity or hemolytic activity poses a significant limitation to their further development (Supplementary Table S3). Cytotoxicity and hemolysis assays confirmed that Scymicrosin_7–26_ was well-tolerated by three mammalian cell lines (RAW264.7, Beas-2B, HEK293T) and exhibited low hemolytic activity toward human erythrocytes (Figure 3).

Conventional antibiotics typically act on discrete molecular targets in bacteria (such as cell wall synthesis, protein synthesis, or nucleic acid replication); this specific nature renders them susceptible to bacterial evasion through target modification or metabolic bypass pathways (Bucataru and Ciobanasu, 2024). Unlike conventional antibiotics, AMPs often employ multiple mechanisms of action (Luo and Song, 2021; Talandashti et al., 2021; Luo et al., 2024). Its amphipathic structure—featuring both hydrophobic and hydrophilic regions—enables insertion into the membrane, resulting in pore formation or membrane dissolution (Marín-Medina et al., 2016; Lorenzon et al., 2019; Zhang et al., 2022). In addition to membrane disruption, AMPs can penetrate the cell membrane to target various intracellular components, and these two mechanisms act in concert (Li et al., 2023; Bucataru and Ciobanasu, 2024). Our findings indicate that Scymicrosin_7–26_, like other typical cationic antimicrobial peptides, exhibits a multimodal antibacterial mechanism involving both membrane-targeting and non-membrane pathways. Membrane disruption was confirmed through PI/SYTO9 staining, NPN uptake assays, and electron microscopy, which revealed substantial damage to bacterial envelope integrity. Beyond membrane permeabilization, Scymicrosin_7–26_ also bound to bacterial genomic DNA, suggesting a potential role in impairing DNA replication and transcription. Moreover, the peptide induced ROS accumulation in bacteria, which may contribute to oxidative damage of proteins, lipids, and nucleic acids, ultimately triggering programmed cell death (Figure 4).

Upon bacterial infection, Gram-negative bacteria release key virulence factors such as LPS, while Gram-positive bacteria shed essential pathogenic components including peptidoglycan and teichoic acids. The immune system recognizes these pathogen-associated molecular patterns (PAMPs) and initiates a coordinated series of host defense responses. In this process, macrophages play a pivotal role in both innate and adaptive immunity through the secretion of multiple cytokines (Håversen et al., 2002). We observed that Scymicrosin_7–26_ significantly attenuated LPS-induced inflammation in RAW264.7 macrophages by suppressing the expression of IL-1β, IL-6, TNF-*α*, iNOS, and COX-2 at both transcriptional and protein levels (Figure 5). Antimicrobial peptides can mitigate inflammatory responses through multiple pathways, including direct LPS binding, immunomodulation, and structural optimization. To investigate its mechanism, we first assessed whether Scymicrosin_7–26_ could neutralize LPS. The Limulus Amebocyte Lysate (LAL) assay showed no neutralization of LPS by the peptide within the concentration range of 1.5–48 μM. Subsequent cellular penetration assays, however, revealed that Scymicrosin_7–26_ (1.5–12 μM) could traverse the cell membrane and enter the cytoplasm. These findings suggest that its anti-inflammatory activity may be mediated primarily through intracellular targets rather than direct LPS neutralization. Further mechanistic investigations revealed that the peptide curbed intracellular ROS generation and inhibited the activation of the MAPK and NF-κB signaling pathways, two central regulators of inflammatory responses (Figures 6, 7).

While prior studies have preliminarily confirmed the antimicrobial activity of the peptide Scymicrosin_7–26_ (Zhou et al., 2025), the present study focuses specifically on clinically isolated multidrug-resistant strains. Furthermore, we have expanded the bacterial panel and employed a broader range of methodologies to provide a more comprehensive evaluation of its antibacterial properties. This study first demonstrated the anti-inflammatory efficacy of the peptide in an *in vitro* inflammation model, accompanied by a preliminary investigation into its mechanism of action. The antimicrobial and anti-inflammatory properties of therapeutic agents generally function not in isolation but through complementary mechanisms that synergistically combat infection. Direct bactericidal activity rapidly reduces pathogen load, while anti-inflammatory action helps modulate host immune responses, thereby preventing excessive activation and subsequent tissue damage. The dual functionality of Scymicrosin_7–26_ suggests its potential therapeutic relevance in complex infections such as sepsis, pneumonia, and infected wounds. It should be noted, however, that this study has certain limitations. The absence of *in vivo* data restricts the translational relevance of the findings, and the conclusions are largely derived from a limited number of bacterial strains, which may introduce bias. Further validation in animal models, along with an expanded panel of clinical isolates, is required to more accurately elucidate the peptide’s activity and potential under physiological conditions.

## 5 Conclusion

In summary, the antimicrobial peptide Scymicrosin_7–26_ demonstrates broad-spectrum activity in vitro against clinically prevalent multidrug-resistant bacteria. It retains efficacy under physiological ion concentrations as well as in the presence of fetal bovine serum (FBS), and shows no antagonism when combined with conventional antibiotics. Notably, Scymicrosin_7–26_ exhibits a low propensity for resistance induction and effectively disrupts both developing and mature biofilms. The peptide also displays favorable biosafety, with low cytotoxicity and hemolytic activity. Mechanistically, Scymicrosin_7–26_ targets both bacterial membrane integrity and intracellular components, and attenuates LPS-induced inflammation by mitigating oxidative stress and suppressing the MAPK and NF-κB signaling pathways. Collectively, these results support the further investigation of Scymicrosin_7–26_ as a candidate worth evaluating in the context of multidrug-resistant bacterial infections.

## Data Availability

The original contributions presented in the study are included in the article/Supplementary material, further inquiries can be directed to the corresponding authors.
