# Supplementary material for: Scymicrosin7–26, a Scylla paramamosain-derived novel antimicrobial peptide, exhibits efficacy against multidrug-resistant ESKAPE pathogens and anti-inflammatory activity
Source: Front Microbiol. 2025 Dec 17;16:1732053. doi: 10.3389/fmicb.2025.1732053 (PMC12753993; doi:10.3389/fmicb.2025.1732053)
Supplement: Supplementary file 1 [file Table_1.DOCX]

Supplementary Material

# Supplementary Figures and Tables

## Supplementary Figures


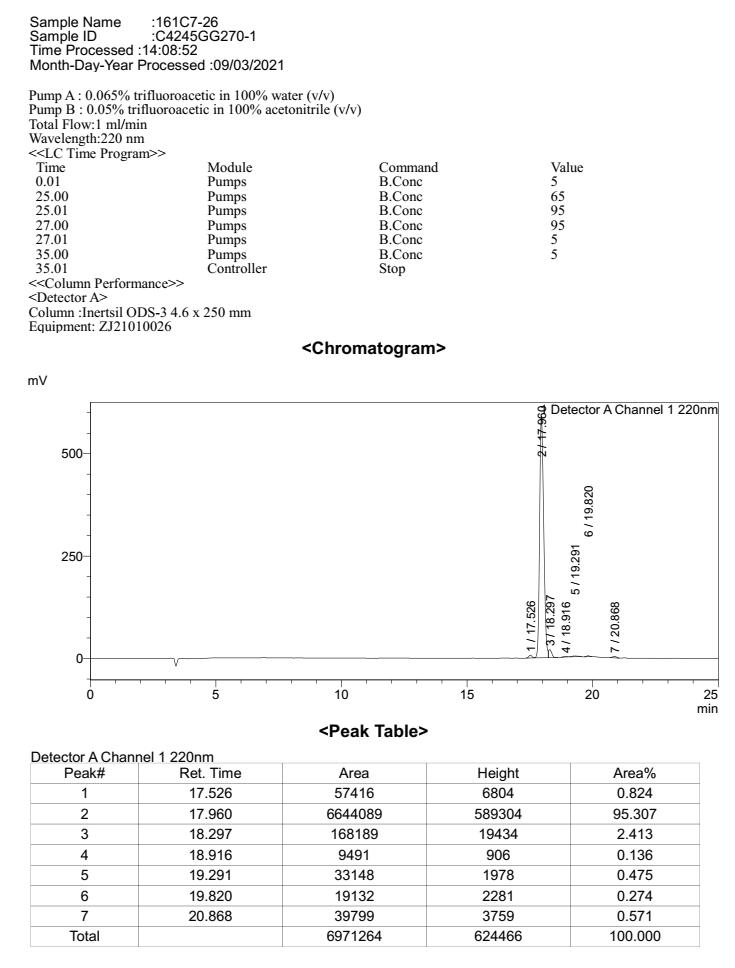


**Figure S1.** The HPLC chromatogram of antimicrobial peptide Scymicrosin7-26.


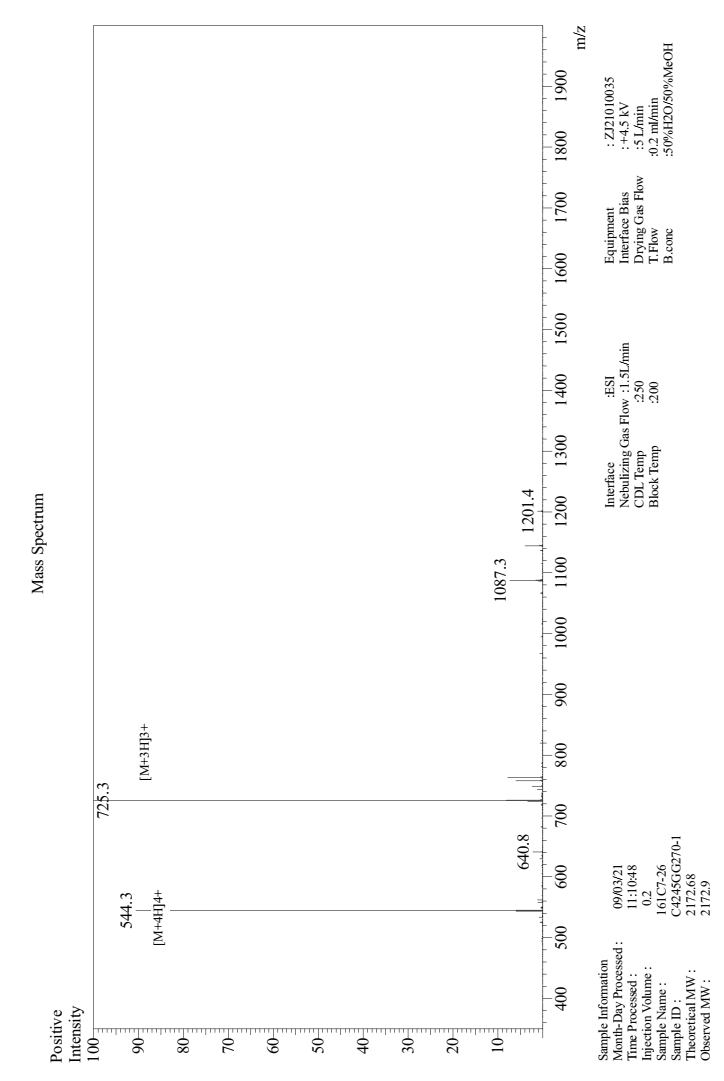


**Figure S2.**The mass spectrum of antimicrobial peptide Scymicrosin7-26.


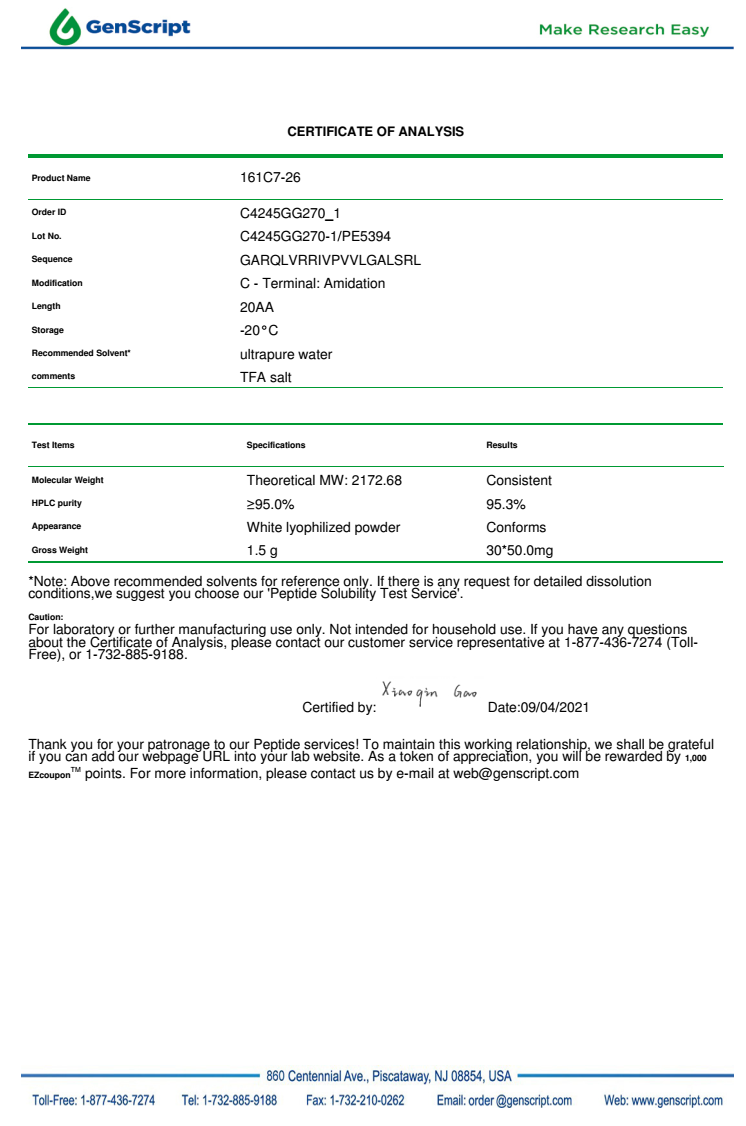


**Figure S3.** The certificate of analysis of the antimicrobial peptide Scymicrosin7-26.


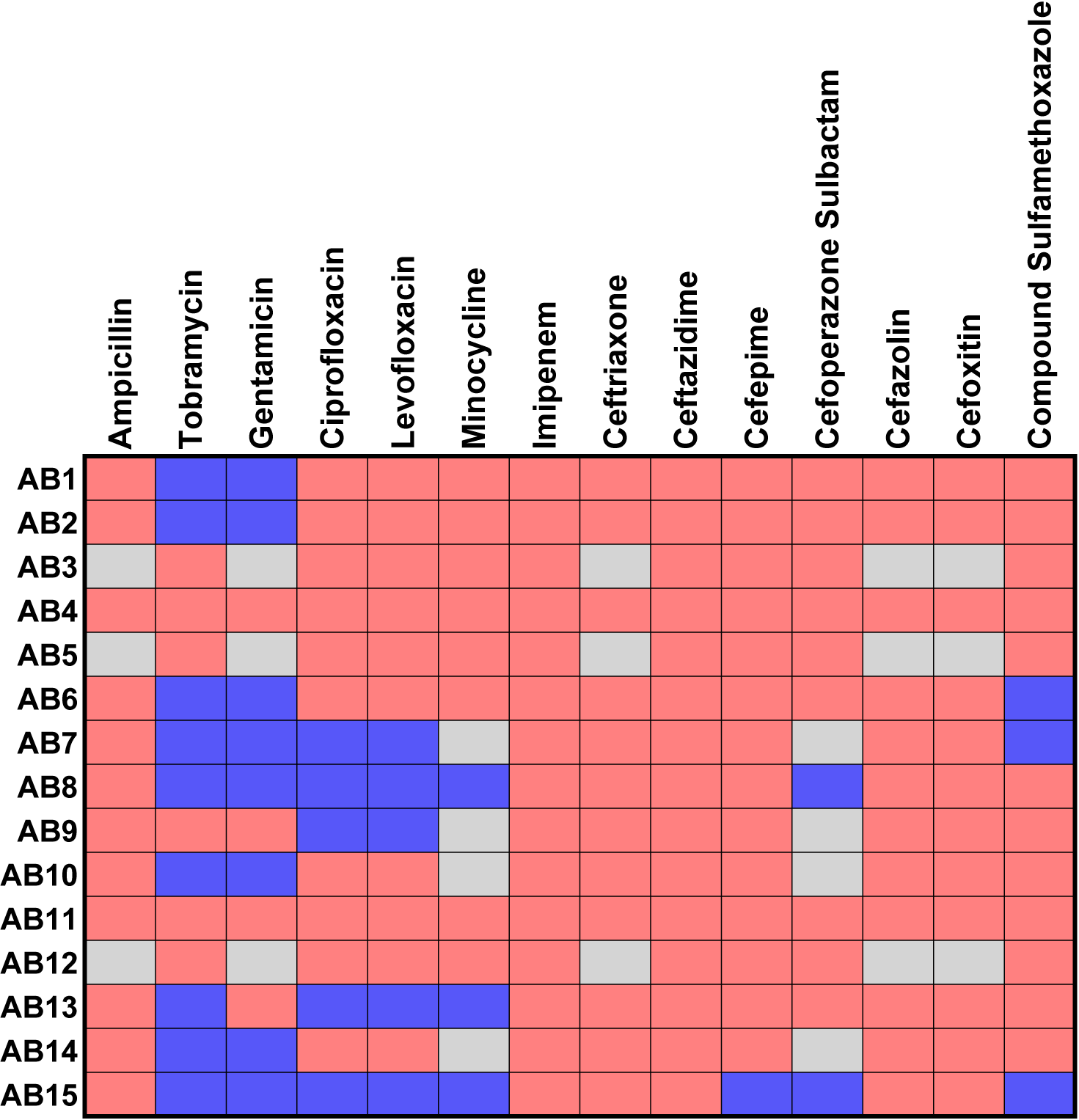


**Figure S4.** **Antibiotic susceptibility profile of multidrug-resistant *Acinetobacter baumannii* isolates.** Red: Intermediate/Resistant; Blue: Susceptible; Gray: Not Tested.

**
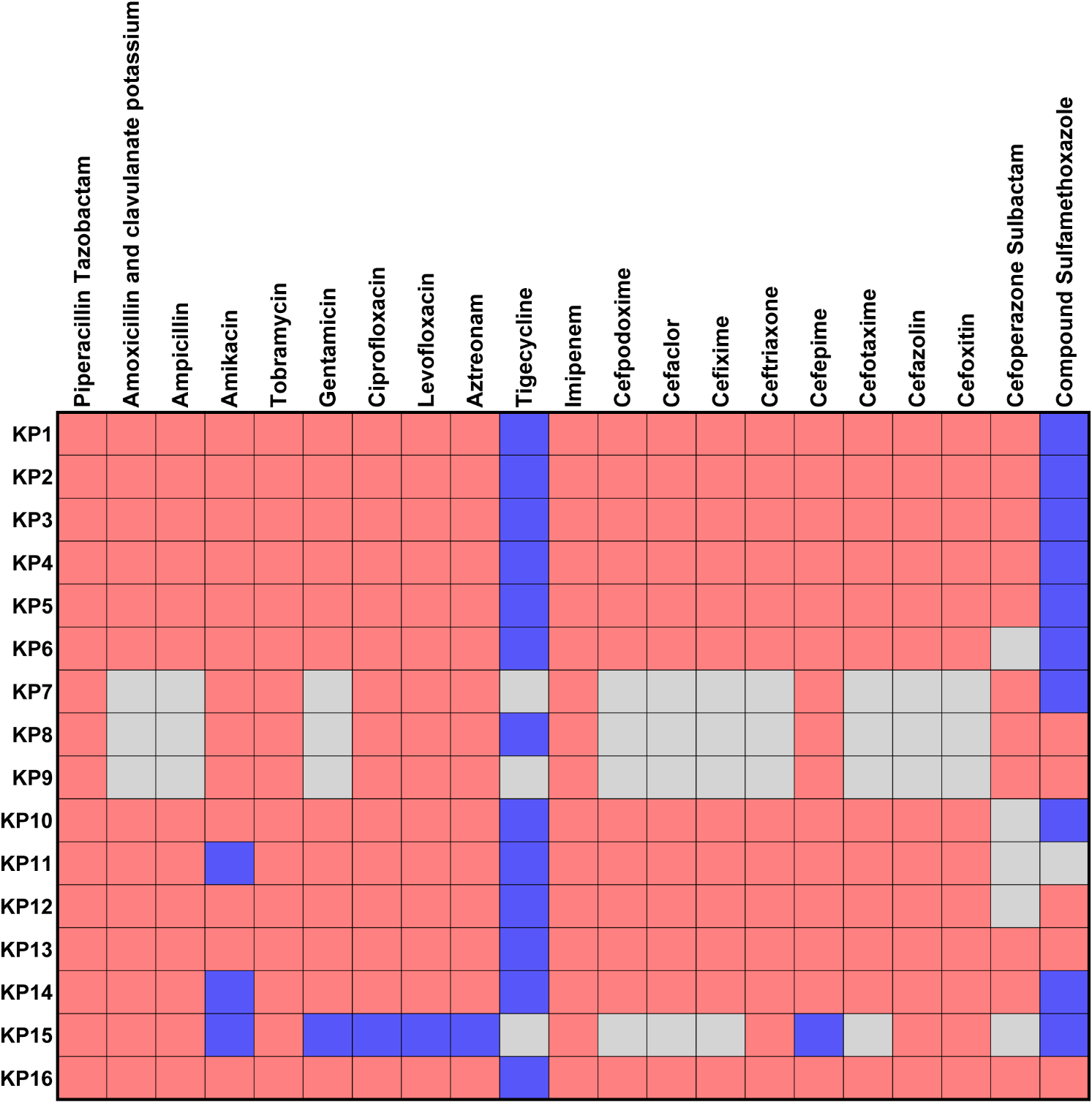
**

**Figure S5.** **Antibiotic susceptibility profile of multidrug-resistant *Klebsiella pneumoniae* isolates.** Red: Intermediate/Resistant; Blue: Susceptible; Gray: Not Tested.


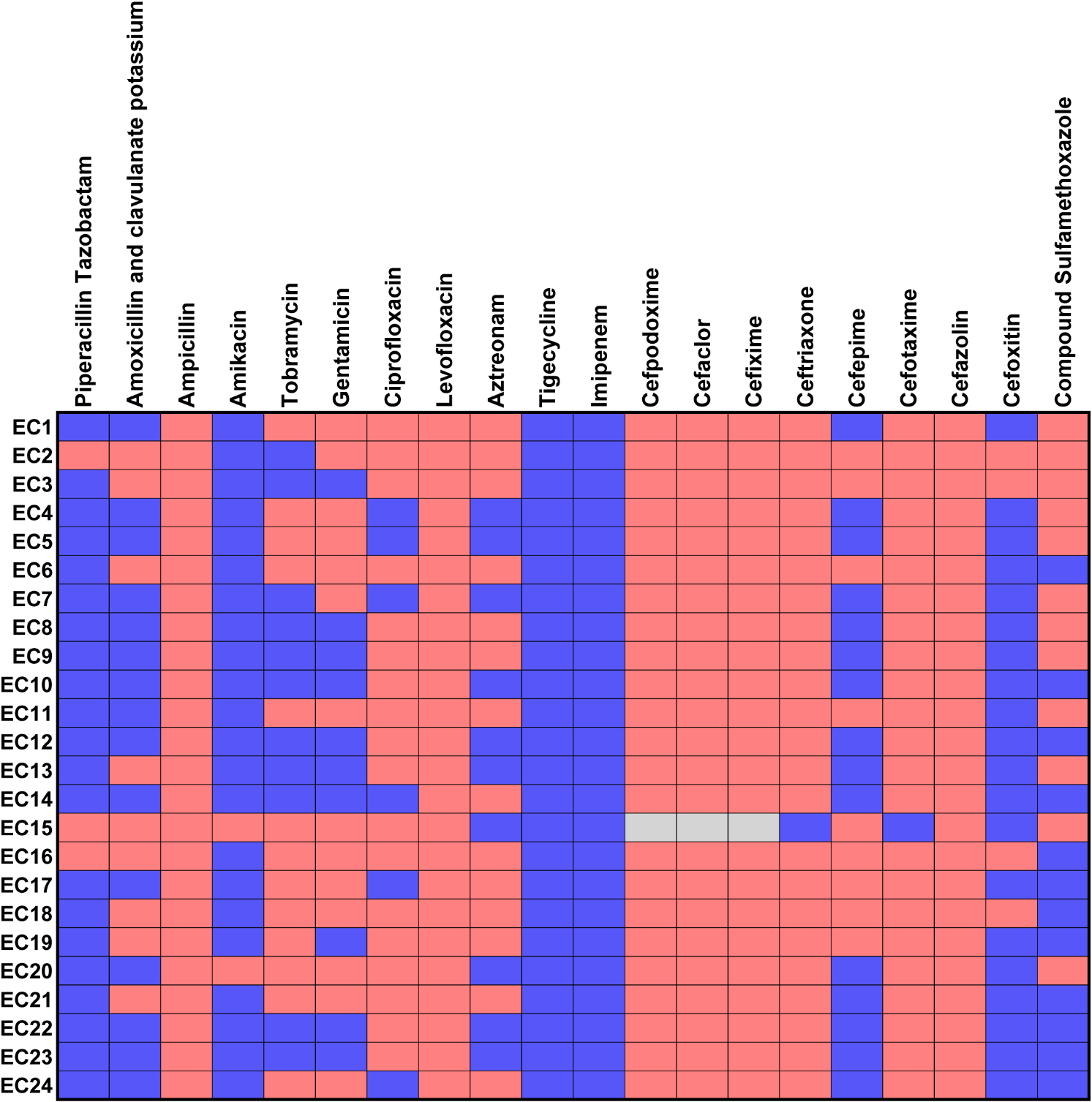


**Figure S6.** **Antibiotic susceptibility profile of multidrug-resistant *Escherichia coli* isolates.** Red: Intermediate/Resistant; Blue: Susceptible; Gray: Not Tested.


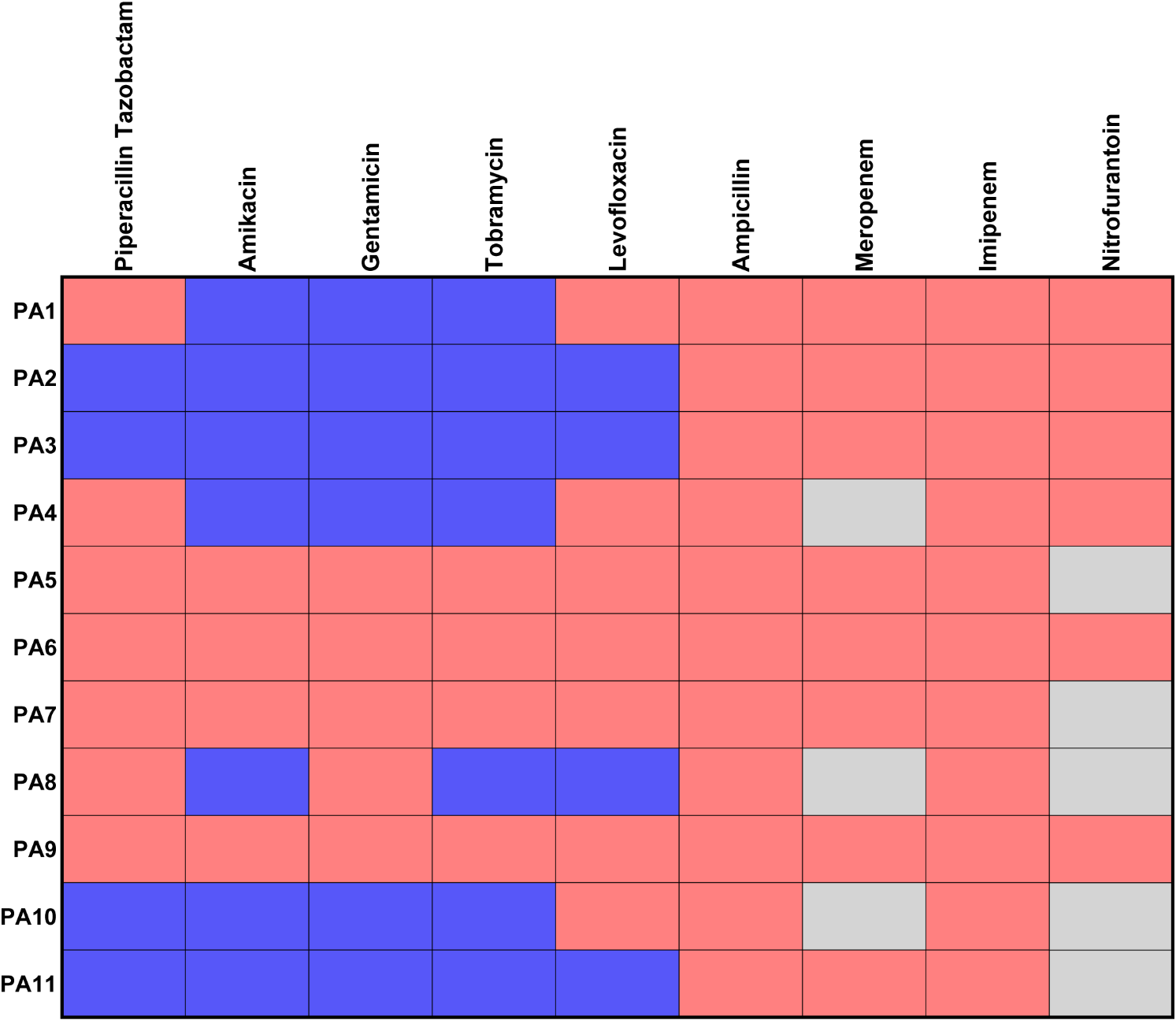


**Figure S7.** **Antibiotic susceptibility profile of multidrug-resistant *Pseudomonas aeruginosa* isolates.** Red: Intermediate/Resistant; Blue: Susceptible; Gray: Not Tested.


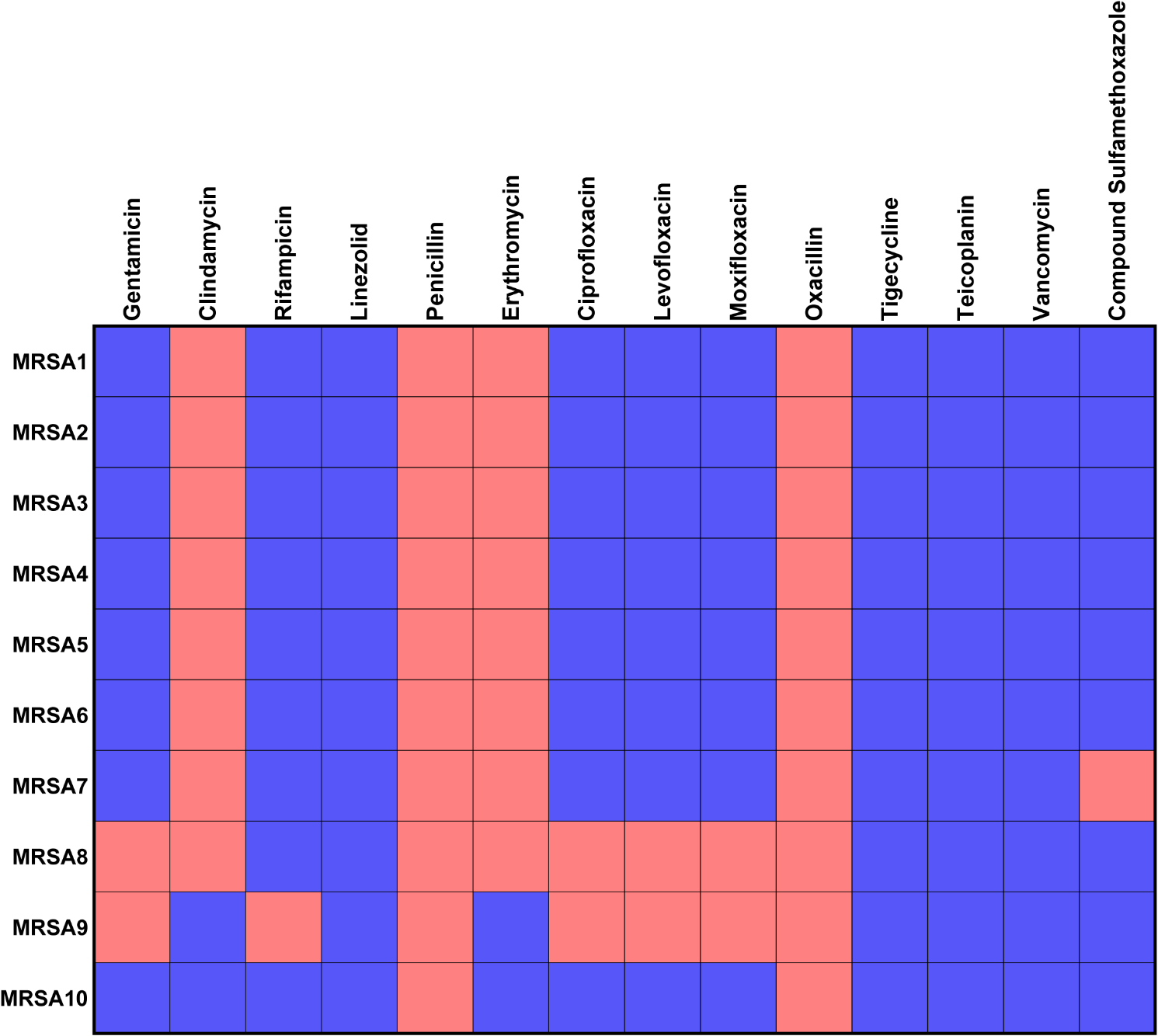


**Figure S8.** **Antibiotic susceptibility profile of methicillin-resistant *Staphylococcus aureus* isolates.** Red: Intermediate/Resistant; Blue: Susceptible.


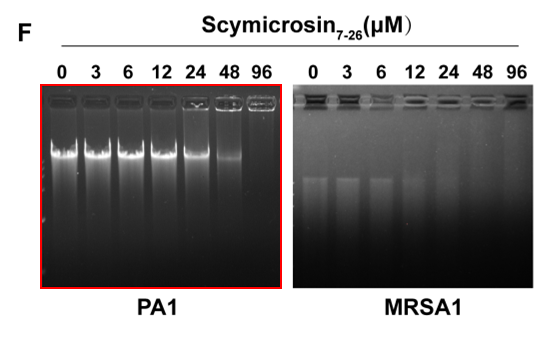

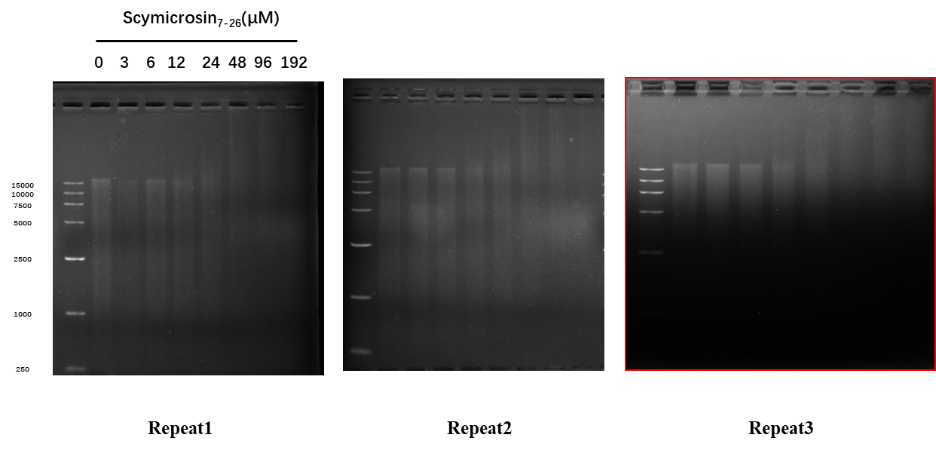


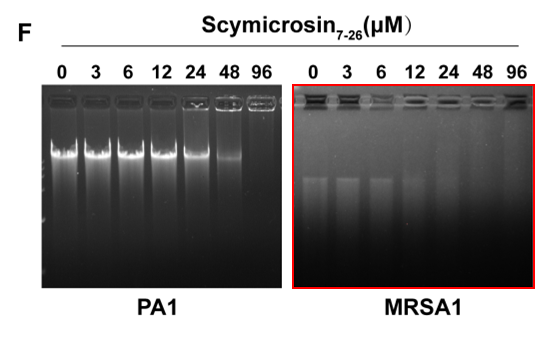

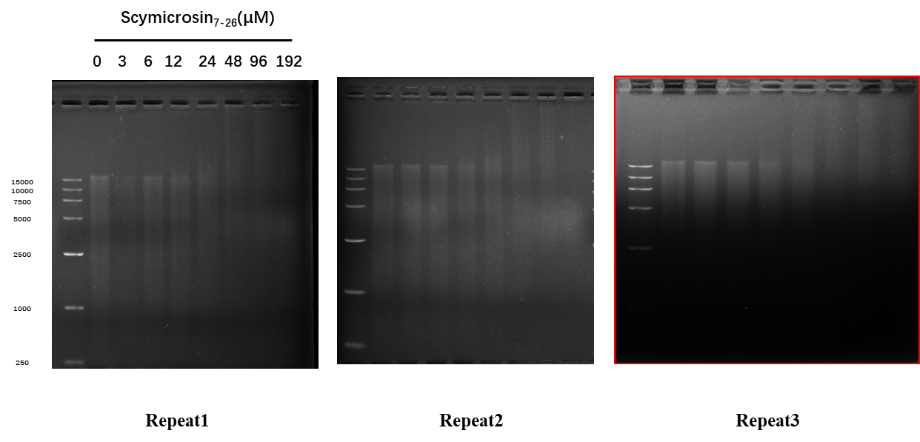


**Figure S9.** Raw gel image corresponding to Fig. 4F.

| PA1 | | MRSA1 | |
| --- | --- | --- | --- |
| Control | 1×MIC | Control | 1×MIC |
| **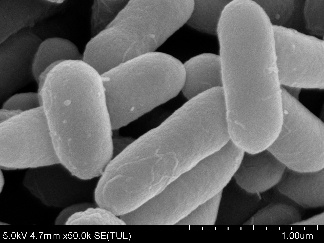** | 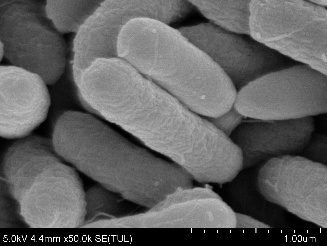 | 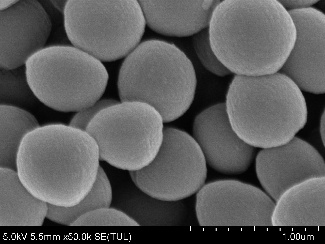 | 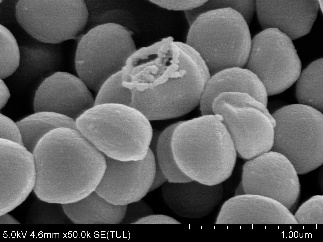 |
| 2×MIC | Polymyxin B | 2×MIC | Lysostaphin |
| **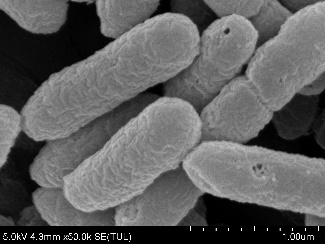** | 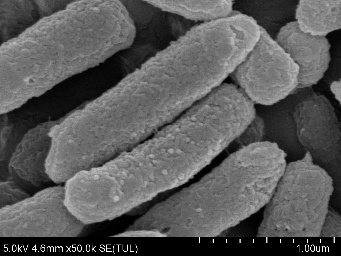 | 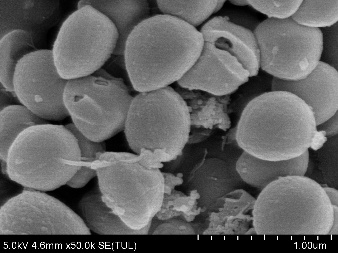 | 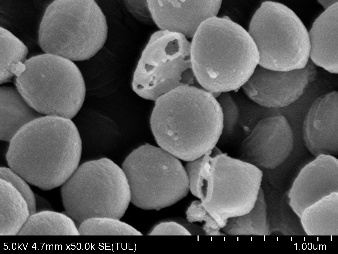 |

**Figure S10.** Raw SEM images of PA1 and MRSA1 strains.

| PA1 | | MRSA1 | |
| --- | --- | --- | --- |
| Control | 1×MIC | Control | 1×MIC |
| **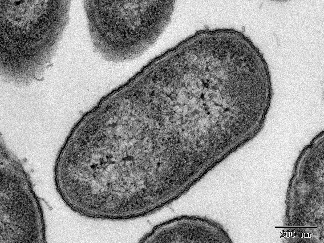** | 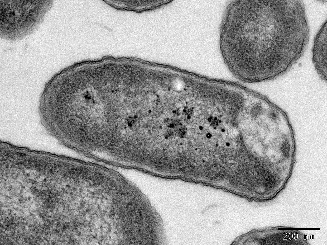 | 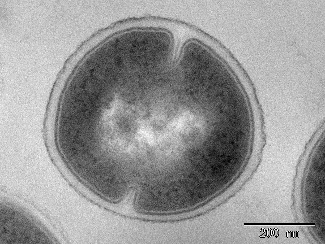 | 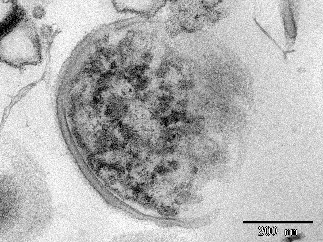 |
| 2×MIC | Polymyxin B | 2×MIC | Lysostaphin |
| **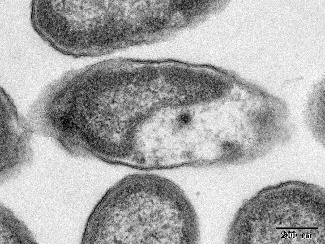** | 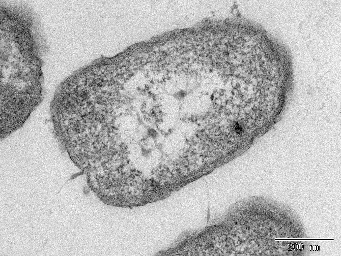 | 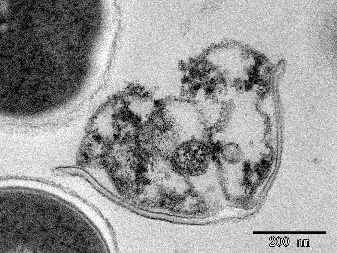 | 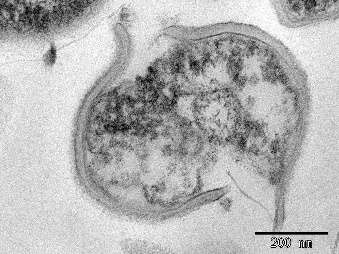 |

**Figure S11.** Raw TEM images of PA1 and MRSA1 strains.

## Supplementary Tables

**Table S1. List of reagents and instruments‌**

| **Reagent /Instruments‌** | **Manufacturer** | **Catalog No.** | **Country** |
| --- | --- | --- | --- |
| LB Broth Medium | Huankai | 028320 | China |
| MH Broth Medium | Huankai | 028040 | China |
| LB Agar Medium | Huankai | 028330 | China |
| Sodium Chloride | Aladdin | C111535 | China |
| Magnesium Chloride | Aladdin | M113687 | China |
| Calcium hloride | Aladdin | C110766 | China |
| Ferric Chloride | Aladdin | I112065 | China |
| Scymicrosin7-26 | Genscript | C4245GG270-1/PE5394 | China |
| Imipenem Hydrate | Solarbio | II1890 | China |
| **Reagent /Instruments‌** | **Manufacturer** | **Catalog No.** | **Country** |
| Clindamycin Hydrochloride | Solarbio | IC0460 | China |
| Polymyxin B Sulfate | Solarbio | IP5100 | China |
| Vancomycin Hydrochloride | Solarbio | IV0030 | China |
| Tigecycline | Solarbio | IT1430 | China |
| Amikacin Sulfate | Solarbio | IA0211 | China |
| Hoechst 33342 | Beyotime | C1022 | China |
| LIVE/DEAD™ BacLight™ Bacterial Viability Kits | Thermo Fisher Scientific | L13152 | USA |
| N-Phenyl-1-naphthylamine (NPN) | Aladdin | P110559 | China |
| 4-(2-Hydroxyethyl)-1-piperazineethanesulfonic Acid (HEPES) | Solarbio | H8090 | China |
| Phosphate-Buffered Saline (PBS) Powder | Solarbio | P1010 | China |
| Anti fluorescence quenching sealing agent | Solarbio | S2100 | China |
| **Reagent /Instruments‌** | **Manufacturer** | **Catalog No.** | **Country** |
| Glutaraldehyde | Aladdin | G105907 | China |
| Nitric Oxide Detection Kit | Beyotime | S0021S | China |
| Bacterial Genomic DNA Extraction Kit | Foregene | DE-05311 | China |
| CCK-8 | Dojindo | CK04 | Japan |
| Triton X-100 | Solarbio | IT9100 | China |
| Crystal Violet | Solarbio | C8470 | China |
| Fetal Bovine Serum (FBS) | Gibco | 10099141c | Australia |
| High Glucose Dulbecco's Modified Eagle Medium (DMEM) | Sigma | D0822 | USA |
| Lipopolysaccharide (LPS) | Sigma | L4391 | USA |
| LPS Detection Kit | Beyotime | C0273S | China |
| TRIzol | Vazyme | r401-01 | China |
| **Reagent /Instruments‌** | **Manufacturer** | **Catalog No.** | **Country** |
| TNF-α ELISA Kit | MultiScience | EK282 | China |
| IL-6 ELISA Kit | MultiScience | EK206 |  |
| IL-β ELISA Kit | MultiScience | EK201B |  |
| Tubulin - β Monoclonal Antibody | Beyotime | AF1216 | China |
| p-p38 Monoclonal Antibody | Zenbio | 310091 | China |
| p38 Monoclonal Antibody | Zenbio | R25239 | China |
| p - ERK Monoclonal Antibody | Zenbio | 301245 | China |
| ERK Monoclonal Antibody | Zenbio | R22685 | China |
| p - JNK Monoclonal Antibody | Zenbio | 340810 | China |
| JNK Monoclonal Antibody | Zenbio | R24780 | China |
| NF - κB P65 Monoclonal Antibody | Zenbio | R25149 | China |
| **Reagent /Instruments‌** | **Manufacturer** | **Catalog No.** | **Country** |
| p - p65 Monoclonal Antibody | Zenbio | 310013 | China |
| IkBα Monoclonal Antibody | Zenbio | R23322 | China |
| p - IkBα Monoclonal Antibody | Zenbio | 340776 | China |
| iNOS Polyclonal Antibody | Zenbio | 340668 | China |
| COX - 2 Monoclonal Antibody | Zenbio | R23971 | China |
| Reverse Transcription Kit | ABM | G237 | Canada |
| ROS Test Kit | Beyotime | S0033S | China |
| PVDF membranes | Millipore | IPVH00010 | USA |
| Cy3-conjugated secondary antibody | Abclonal | AS007 | China |
| RAW264.7 cells | Procell | CL-0190 | China |
| Beas-2B cells | Sunncell | SNL-203 | China |
| **Reagent /Instruments‌** | **Manufacturer** | **Catalog No.** | **Country** |
| HEK293T cells | Procell | CL-0005 | China |
| 4% human erythrocyte | Jingkebio, | JKA1150 | China |
| Real - time Fluorescent Quantitative PCR Kit | Transgen | AQ602-01 | China |
| Multifunctional microplate reader (SpectraMax i3x) | Molecular Devices | SpectraMax i3x | USA |
| Applied Biosystems Real-Time PCR System | Thermo Fisher Scientific | ABI 7500 | USA |
| Chemiluminescence Imaging Analysis System | Bio-rad | ChemiDoc Touch | USA |
| Inverted fluorescence microscope | Leica | DMi8 | Germany |

**Table S2. List of Abbreviations**

| **Abbreviation** | **Full Name** |
| --- | --- |
| AMPs | Antimicrobial peptides |
| MIC | Minimum inhibitory concentration |
| MBC | minimum bactericidal concentration |
| PI | Propidium iodide |
| NPN | N-Phenyl-1-naphthylamine |
| CCK-8 | Cell Counting Kit-8 |
| TNF-α | Tumor necrosis factor |
| IL-1β | Interleukin-1β |
| IL-6 | Interleukin-6 |
| NO | Nitric oxide |
| COX-2 | Cyclooxygenase-2 |
| iNOS | Inducible nitric oxide synthase |
| qRT-PCR | Quantitative real-time PCR |
| WB | Western blot |
| NF-κB | Nuclear factor κB |
| MAPK | Mitogen-activated protein kinase |
| IκBα | Inhibitor of κBα |
| JNK | c-Jun N-terminal kinase |
| Fetal bovine serum | Extracellular regulated protein kinase |
| **Abbreviation** | **Full Name** |
| LPS | Lipopolysaccharide |
| HEPES | 4-(2-Hydroxyethyl)-1-piperazineethanesulfonic acid |
| FBS | Fetal bovine serum |
| ELISA | Enzyme-linked immunosorbent assay |

**Table S3. Comparative Profiling Table of Various Antimicrobial Peptides**

| **Peptides** | **benchmarking sequence** | **net charge** | **Hydrophobicity (%)** | **MIC ranges on ESKAPE species** | **biofilm effects** | **hemolysis** | **salt tolerance** | **anti-inflammatory readouts** | **Type** | **Source / Design** |
| --- | --- | --- | --- | --- | --- | --- | --- | --- | --- | --- |
| KR-12 | KRIVQRIKDFLR | +5 | 41% | *E. coli* K12 : 40 uM  MRSA-inactive | N/R | 10% hemolysis at  100μM (hRBC) | NaCl-sensitive:E. coli  (100 mM). | N/R | Synthetic | LL-37 |
| SAAP-148 | LKRVWKRVFKLLKRYWRQLKKPVR | +11 | 41% | LC₉₉.₉ in 50% plasma  E. faecium LUH15122: 6.4 uM  S. aureus LUH14616: 3.2 - 12.8 uM  K. pneumoniae LUH15104: 12.8 - 25.6 uM  A. baumannii RUH875: 1.6 - 3.2 uM  P. aeruginosa LUH15103: 12.8 - 25.6 uM  E. cloacae LUH15114: 3.2 - 12.8 uM  E. coli LUH15117: 6.4 uM | S. aureus JAR060131: 81% ( plasma-coated)  and 72% (uncoated) eradication at 12.8μM  A. baumannii RUH875: 85% (plasma-coated) eradication  at 12.8μM | 50% hemolysis at  ＜~10μM (hRBC) | N/R | N/R | Synthetic | LL-37 |
| AR-23 | AIGSILGALAKGLPTLISWIKNR | +4 | 52% | *E.coli*:20-25 uM  *S. aureus*：3.5 uM  *K. pneumoniae* ATCC 10031 : 6.25 uM | N/R | 50% hemolysis at  8μM (hRBC) | N/R | N/R | Natural | Rana tagoi, Asia  melittin-related peptide |
| Melectin | GFLSILKKVLPKVMAHMK | +5 | 55% | E. coli : 2-25 uM  MRSA : 6.8-25 uM  P. aeruginosa : 18.5 uM | N/R | 50% hemolysis at  ＞100μM (rat RBC) | N/R | N/R | Natural | Cleptoparasitic bee, Melecta albifrons |
| Anisaxin-1 | SWLSKTYKKLENSAKKRIAEGIAIALRGGPR | +6 | 35% | E. coli ATCC, c.i. or c.i.2 : 1-2 uM  S. aureus ATCC, c.i., or c.i.2 : 16-32 or＞64uM  K. pneumoniae ATCC, c.i., c.i.2 : 0.5-4 uM  A. baumannii ATCC, c.i., or c.i.2. : 1 uM  P. aeruginosa ATCC, c.i., or c.i.2 : 4-8 uM | N/R | 50% hemolysis at  78μM (hRBC) | N/R | N/R | Natural | Anisakis pegreffii  cecropins |
| Anisaxin-2S | SWLSKTWKKLENSGKKRIAEGIAIALKGGLR | +6 | 39% | E. coli ATCC, c.i. or c.i.2 : 0.5-1 uM  S. aureus ATCC : 4 uM  S. aureus c.i., or c.i.2 : ＞64 uM  K. pneumoniae ATCC, c.i., c.i.2 : 0.25-1 uM  A. baumannii ATCC, c.i., or c.i.2. : 0.25-0.5 uM  P. aeruginosa ATCC, c.i., or c.i.2 : 1-8 uM | N/R | N/R | N/R | N/R | Natural | Anisakis pegreffii  cecropins |
| Anisaxin-2P | SWLSKTWKKLENSGKKRIAEGIAIALKGGAR | +6 | 39% | E. coli ATCC, c.i. or c.i.2 : 0.5-2 uM  S. aureus ATCC : 32-64 uM  S. aureus c.i., or c.i.2 : ＞64 uM  K. pneumoniae ATCC, c.i., c.i.2 : 0.5-2 uM  A. baumannii ATCC, c.i., or c.i.2. : 0.5-1 uM  P. aeruginosa ATCC, c.i., or c.i.2 : 4-16 uM | N/R | N/R | N/R | N/R | Natural | Anisakis pegreffii  cecropins |
| Scymicrosin_7‑26_ | GARQLVRRIVPVVLGALSRL-NH₂ | +4 | 52.6% | MDR *Escherichia coli* : 3-12 μM  MDR *P.aeruginosa* : 6-48 μM  MDR *K.pneumoniae* : 6-12 μM  MDR *A. baumannii* : ≤6 μM  MRSA : 3-6 μM | MRSA1: 100% inhibition at 6 μM,  92.8% eradication at 12 μM;  PA1: 100% inhibition at 24 μM,  31.7% eradication at 24 μM | 7.6% hemolysis at  48 μM (hRBC) | EC1：NaCl: 2× MIC  Cacl_2_:8× MIC  Fecl3:1× MIC  PA1：NaCl: 1× MIC  Cacl_2_:＞8× MIC  Fecl3:1× MIC  KP1：NaCl: 4× MIC  Cacl_2_:2× MIC  Fecl3:1× MIC  AB1：NaCl: 1× MIC  Cacl_2_:8× MIC  Fecl3:1× MIC  MRSA1：NaCl: 2× MIC  Cacl_2_:4× MIC  Fecl3:1× MIC | Active from 3 μM | Synthetic | Scylla paramamosain |

All data above are from the APD3 Antimicrobial Peptide Database, accessible at: https://aps.unmc.edu/. Data updated through September 10, 2025. c.i.:clinical isolate.

**Table S4.** **Primers used in this study**

| Gene Name | Forward（5´－3´） | Reverse （5´－3´） |
| --- | --- | --- |
| GAPDH | TCACCACCATGGAGAAGGC | GCTAAGCAGTTGGTGGTGCA |
| TNF-α | CCTCACCCACACCGTCAG | GTTGGTCCCCCTTCTCCA |
| IL-1β | GAAATGCCACCTTTTGACAGTG | TGGATGCTCTCATCAGGACAG |
| IL-6 | CTGCAAGAGACTTCCATCCAG | AGTGGTATAGACAGGTCTGTTGG |
| INOS | CTGCAGCACTTGGATCAGGAACCTG | GGGAGTAGCCTGTGTGCACCTGGAA |

**Table S5. Results of Combination Use of Scymicrosin7‑26 with Other Drugs**

| **Strain Drug** | **MIC** | | **FICI** | **Result** |
| --- | --- | --- | --- | --- |
|  | **Alone** | **In combination** |  |  |
| ***A.baumannii* (AB1)** |  |  |  |  |
| Scymicrosin_7‑26_（μM） | 3 | 1.5 | 0.625 | Additive |
| Amikacin（ug/ml） | 2 | 0.25 |  |  |
| Scymicrosin_7‑26_（μM） | 3 | 1.5 | 0.625 | Additive |
| Imipenem（ug/ml） | 64 | 8 |  |  |
| Scymicrosin_7‑26_（μM） | 3 | 1.5 | 0.563 | Additive |
| Tigecycline（ug/ml） | 1 | 0.0625 |  |  |
| Scymicrosin_7‑26_（μM） | 3 | 0.0234 | 1.008 | Indifferent |
| Polymyxin B（ug/ml） | 4 | 4 |  |  |
| ***E. coli* (EC1)** |  |  |  |  |
| Scymicrosin_7‑26_（μM） | 6 | 0.0469 | 0.508 | Additive |
| Amikacin（ug/ml） | 4 | 2 |  |  |
| Scymicrosin_7‑26_（μM） | 6 | 1.5 | 0.5 | Synergistic |
| Imipenem（ug/ml） | 0.125 | 0.03125 |  |  |
| Scymicrosin_7‑26_（μM） | 6 | 3 | 1 | Additive |
| Tigecycline（ug/ml） | 0.125 | 0.0625 |  |  |
| Scymicrosin_7‑26_（μM） | 6 | 3 | 0.625 | Additive |
| Polymyxin B（ug/ml） | 4 | 0.5 |  |  |
| ***P.aeruginosa* (PA1)** |  |  |  |  |
| Scymicrosin_7‑26_（μM） | 24 | 0.0936 | 1.004 | Indifferent |
| Amikacin（ug/ml） | 16 | 16 |  |  |
| Scymicrosin_7‑26_（μM） | 24 | 12 | 0.625 | Additive |
| Imipenem（ug/ml） | 128 | 16 |  |  |
| Scymicrosin_7‑26_（μM） | 24 | 6 | 0.5 | Synergistic |
| Tigecycline（ug/ml） | 16 | 4 |  |  |
| Scymicrosin_7‑26_（μM） | 24 | 3 | 0.375 | Synergistic |
| Polymyxin B（ug/ml） | 4 | 1 |  |  |
| ***k.pneumoniae* (KP1)** |  |  |  |  |
| Scymicrosin_7‑26_（μM） | 6 | 3 | 0.625 | Additive |
| Amikacin（ug/ml） | 64 | 8 |  |  |
| Scymicrosin_7‑26_（μM） | 6 | 1.5 | 0.5 | Synergistic |
| Imipenem（ug/ml） | 32 | 8 |  |  |
| Scymicrosin_7‑26_（μM） | 6 | 0.0234 | 1.004 | Indifferent |
| Tigecycline（ug/ml） | 1 | 1 |  |  |
| Scymicrosin_7‑26_（μM） | 6 | 3 | 1 | Additive |
| Polymyxin B（ug/ml） | 4 | 2 |  |  |
| ***S.aureus* (MRSA1)** |  |  |  |  |
| Scymicrosin_7‑26_（μM） | 6 | 0.0469 | 1.008 | Indifferent |
| Clindamycin（ug/ml） | 0.125 | 0.125 |  |  |
| Scymicrosin_7‑26_（μM） | 6 | 1.5 | 0.375 | Synergistic |
| Imipenem（ug/ml） | 128 | 16 |  |  |
| Scymicrosin_7‑26_（μM） | 6 | 3 | 1 | Additive |
| Tigecycline（ug/ml） | 1 | 0.5 |  |  |
| Scymicrosin_7‑26_（μM） | 6 | 0.0469 | 1.008 | Indifferent |
| Vancomycin（ug/ml） | 2 | 2 |  |  |
